# Effects of FGF21, soluble TGFBR2, and environmental temperature on metabolic dysfunction in lipodystrophic mice

**DOI:** 10.1172/jci.insight.194882

**Published:** 2025-07-15

**Authors:** Jessica N. Maung, Yang Chen, Keegan S. Hoose, Rose E. Adler, Hadla Hariri, Mia J. Dickson, Taryn A. Hetrick, Gabriel A. Ferguson, Rebecca L. Schill, Hiroyuki Mori, Romina M. Uranga, Kenneth T. Lewis, Isabel D.K. Hermsmeyer, Donatella Gilio, Christopher de Solis, Amber Toliver, Noah Davidsohn, Elif A. Oral, Ormond A. MacDougald

**Affiliations:** 1 Department of Molecular & Integrative Physiology, University of Michigan Medical School, Ann Arbor, Michigan, USA.; 2 Department of Internal Medicine, University of Michigan Medical School, Ann Arbor, Michigan, USA.; 3 Rejuvenate Bio, San Diego, California, USA.

**Keywords:** Bone biology, Metabolism, Adipose tissue, Bone marrow, Obesity

## Abstract

Metabolic health is influenced by adipose tissue, and obesity and lipodystrophy are characterized by inflammation and metabolic dysfunction. Whereas obesity and lipodystrophy treatments involve pharmacological approaches and lifestyle changes, these therapies require long-term, repeated dosing and are not successful for all patients. Gene therapy with targets such as FGF21 and soluble TGF-β receptor 2 (sTGFBR2) provides an alternative approach, specifically in lipodystrophy. Preclinical experiments in mice housed at 22°C are confounded by a mild cold stress not generally experienced by humans, which can negatively affect translation of metabolic therapeutics. In this study, we investigated effects of FGF21/sTGFBR2 combination gene therapy on obese and lipodystrophic mice and how housing temperature influences therapeutic efficacy. In obese mice, FGF21/sTGFBR2 improved insulin resistance and hyperlipidemia more dramatically at warmer temperatures. In lipodystrophic mice on a high-fat diet, combination therapy required adipose tissue to improve insulin resistance at 30°C, whereas FGF21 alone improved insulin resistance at 22°C. Transcriptomic analyses revealed that lipodystrophic mice had upregulated hepatic cell proliferation and fibrosis pathways and that FGF21 promoted hepatic metabolism. Thus, metabolic dysfunction caused by lipodystrophy is improved by targeting FGF21 and TGFB signaling, but effectiveness in preclinical models may be dependent upon environmental temperature and presence of adipose tissue.

## Abstract 2

<span>FGF21/sTGBFR2 gene therapy improves insulin resistance and hyperlipidemia in preclinical models of obesity and lipodystrophy, but effectiveness in these models are dependent on environmental temperature.</span>

## Introduction

Metabolic health is largely dictated by the amount, location, and function of adipose tissues. Obesity is a medical condition characterized by an excessive accumulation of body fat and often is estimated clinically with waist-to-hip ratio and other measurements (1). It has become a global health concern, with prevalence rates of obesity rising steadily — current estimates are that more than 600 million adult individuals have obesity (2). Obesity is linked to a range of metabolic dysfunctions, including insulin resistance, hypertension, dyslipidemia, and an increased risk of type 2 diabetes, heart disease, and certain cancers (3). At the other extreme, lipodystrophy syndromes are rare, heterogeneous disorders characterized by partial or complete loss of adipose tissues (4). Whereas clinical prevalence of lipodystrophy is about 1 in 20,000 individuals, its genetic prevalence may be as high as 1 in 7,000 (5, 6). With a paucity of adipose tissue in which to store excess energy, lipids instead accumulate ectopically in tissues such as liver, muscle, and pancreas, resulting in metabolic dysfunctions similar to those seen in obesity, including insulin resistance, dyslipidemia, and fatty liver disease (7).

Current treatments for both obesity and lipodystrophy often include pharmacological approaches in addition to lifestyle modifications. For obesity, drugs such as glucagon-like peptide-1 (GLP-1) receptor agonists promote satiety, reduce appetite, and enhance insulin secretion to stimulate weight loss, improve metabolism, and reduce cardiovascular disease (8–11). However, these drugs can also have side effects, including gastrointestinal perturbations and hypotension (12). Patients with lipodystrophy often have low concentrations of leptin in circulation, and leptin replacement therapy is used to manage metabolic dysfunctions associated with leptin deficiency and hyperphagia (13–15). Recombinant leptin therapy helps improve insulin sensitivity, reduce circulating triglyceride concentrations, and decrease liver volume in patients with lipodystrophy; however, leptin therapy does not adequately address all the metabolic perturbations for reasons that are incompletely understood (13). Treatment of metabolic dysfunctions associated with both obesity and lipodystrophy is typically long-term, with some therapies potentially being lifelong. Leptin replacement therapy requires daily subcutaneous injections, whereas GLP-1 receptor agonists are administered at least once weekly. Novel therapies with an extended duration of action and reduced frequency of administration are crucial for improving patient compliance and outcomes, in addition to providing alternative therapies for patients in which current treatments are ineffective or too expensive. Gene therapy, which offers the potential for sustained therapeutic effects, represents a promising approach for providing long-term treatment solutions specifically for lipodystrophy (16).

In recent years, 2 proteins delivered via gene therapy have shown potential for treatment of metabolic dysfunction. First, FGF21 is an endocrine factor, produced and secreted primarily by the liver, that acts at numerous sites, such as adipose tissues and bone, to regulate energy balance, glucose and lipid metabolism, and insulin sensitivity (17, 18). Second, soluble TGF-β receptor 2 (sTGFBR2) binds to TGFB1 ligands and prevents their interaction with cell surface receptors, thus repressing physiological processes, such as immune response, tissue repair, fibrosis, and cell growth (19, 20). Studies have shown that FGF21 gene therapy reduces body weight, adipose tissue hypertrophy and inflammation, and hepatic steatosis, and improves insulin sensitivity, with sustained effects present for over 1 year in obese mice (21, 22). In mice fed a high-fat diet (HFD), combined gene therapy with FGF21 and sTGFBR2 effectively mitigated 4 age-related diseases: obesity, type 2 diabetes, heart failure, and renal failure (23). Potential of these factors to improve metabolic dysfunction in lipodystrophic mice has also been explored. Although metabolic benefits of FGF21 were dependent on presence of adipose tissue in aP2-SREBP1c lipodystrophic mice, an FGF21 analog improved insulin sensitivity and adipocyte gene expression in seipin-deficient lipodystrophic mice (24, 25). In contrast, inhibition of TGFB signaling more effectively alleviated abnormal glucose and lipid metabolism in aP2-SREBP1c lipodystrophic mice than in obese mice (26). Taken together, combination gene therapy of FGF21 and sTGFBR2 has the potential to improve metabolic conditions associated with lipodystrophy.

It is important to note that FGF21 and sTGFBR2 therapies have been tested in mice housed at ~22°C, a temperature comfortable for humans but below the murine thermoneutral zone of ~30°C (27). Humans typically live within thermoneutral temperatures because of thermostats controlling room temperature and ease of adjusting warmth with clothing (27). Mice undergo nonshivering thermogenesis at standard housing temperatures, resulting in elevated energy expenditure, altered metabolic profiles, and increased food intake and physical activity (28, 29). Mice housed at thermoneutrality also have improved immune response, lower heart rate, and decreased bone mineral density (27, 30). Variations in housing temperatures can lead to differences in baseline physiology and treatment efficacies; a previous study found that the degree of weight loss after treatments like GLP-1 and FGF21 in mice varied based on housing temperatures and that drug efficacy could not be predicted based on housing temperature (31). These data underscore the importance of testing preclinical treatments at a variety of housing temperatures to uncover therapeutic effects that might otherwise be missed. In our study, we investigated effects of housing temperatures of 22°C, 26°C, and 30°C with or without gene therapy administration. The intermediate temperature of 26°C was chosen because many universities utilize 26°C housing rooms as an intermediate between ambient temperature and mouse thermoneutrality, and some strains of mice can have a lower thermoneutral point closer to 26°C, with some researchers affirming that 30°C is too warm for mouse studies (32). Overall, mice prefer to live at environmental temperatures between 26°C and 29°C, even if that is not their physiological thermoneutral point (33).

In the present investigation, our overarching goal was to explore the potential of FGF21/sTGFBR2 gene therapy in improving lipodystrophy-associated metabolic dysfunction. First, to evaluate the best experimental conditions for these studies, we tested effects of 3 housing temperatures on efficacy of FGF21/sTGFBR2 gene therapy in obese mice. Then, we evaluated whether FGF21 alone or FGF21/sTGFBR2 combination therapy improved metabolic dysfunction associated with lipodystrophy in an adipocyte-specific lamin A/C–knockout (*Lmna*^ADKO^) mouse model (34). We were particularly interested in effects of gene therapy on insulin resistance, hepatic steatosis, and bone mass, as these variables have been shown to be altered with FGF21 treatment. In diet-induced obese mice, we found that FGF21/sTGFBR2 had greater improvements on insulin sensitivity and hyperlipidemia at warmer temperatures (i.e., 26°C and 30°C). Furthermore, we found that FGF21/sTGFBR2 gene therapy was dependent on adipose tissue to improve insulin resistance in lipodystrophic mice housed at 30°C, but FGF21 alone improved insulin resistance in lipodystrophic mice at 22°C. FGF21/sTGFBR2 or FGF21 alone was sufficient to reduce hepatic steatosis in lipodystrophic mice, and dual FGF21/sTGFBR2 therapy reduced cortical bone volume fraction. RNA-sequencing (RNA-Seq) revealed that livers of lipodystrophic mice had increased expression of pathways associated with cell proliferation and fibrosis and that FGF21 increased pathways associated with hepatic mitochondrial metabolism and protein translation. Taken together, these experiments suggest that targeting FGF21 and TGFB1 may have utility for treatment of metabolic dysfunction associated with lipodystrophy and that murine housing temperature is an important variable to consider when studying therapeutic metabolic endpoints.

## Results

### FGF21/sTGFBR2 combination therapy reduces body weight, improves insulin sensitivity, and reduces plasma triacylglycerol in HFD-fed mice, with greater effects at 26°C and 30°C than at 22°C.

**Figure 1:**
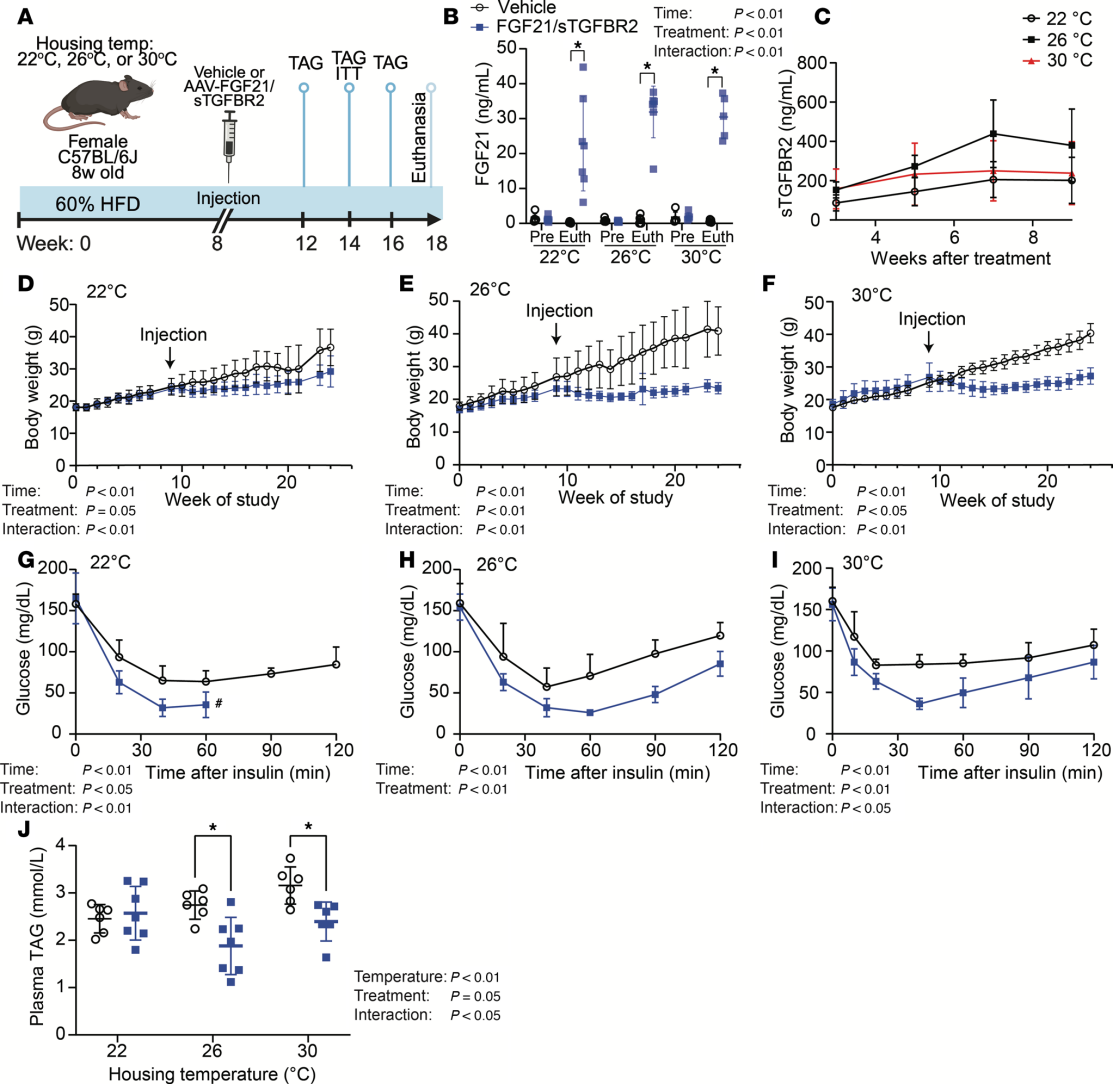
FGF21/sTGFBR2 reduces body weight, improves insulin sensitivity, and reduces plasma TAG in HFD-fed mice, with greatest effects in mice housed at 26°C and 30°C. (**A**) Schematic illustration of housing conditions and experimental timeline. (**B**) Concentrations of FGF21 in plasma pretransduction (Pre) and at euthanasia (Euth). (**C**) Concentrations of sTGFBR2 in plasma 3, 5, 7, and 9 weeks after transduction. Body weight over time in 60% HFD-fed female mice housed at (**D**) 22°C, (**E**) 26°C, or (**F**) 30°C (*n* = 6–7). Insulin tolerance tests (0.75 U/kg) in mice housed at (**G**) 22°C, (**H**) 26°C, or (**I**) 30°C (*n* = 6–7), data presented as mean ± SEM. #Mice in **G** were given intraperitoneal glucose because their blood glucose concentrations dropped below 30 mg/dL. (**J**) Plasma TAG concentrations 8 weeks posttreatment in mice housed at 22°C, 26°C, or 30°C (*n* = 6–7). *P < 0.05. Statistical analyses were performed using 2-way ANOVA, followed by Bonferroni’s post hoc test. TAG, triacylglycerol.

To investigate impacts of environmental temperature on metabolic effects of FGF21/sTGFBR2 in obese mice, 8-week-old female C57BL/6J mice were housed at 22°C, 26°C, or 30°C and maintained on a 60% HFD for 8 weeks. Mice were then administered vehicle, or adeno-associated viruses (AAVs) expressing FGF21 and sTGFBR2 (Figure 1A). FGF21/sTGFBR2 viral transduction elevated circulating FGF21 and sTGFBR2 for the duration of the experiment for all environmental temperatures (Figure 1, B and C). Supraphysiological concentrations of FGF21/sTGFBR2 reduced body weights, with greatest effects observed in mice housed at 26°C or 30°C (Figure 1, D–F). Core body temperature was increased by FGF21/sTGFBR2 therapy in all 3 housing conditions (Supplemental Figure 1, A–C; supplemental material available online with this article; https://doi.org/10.1172/jci.insight.194882DS1). Insulin tolerance tests conducted 6 weeks after transduction with AAV revealed improved insulin sensitivity across all housing temperatures (Figures 1, G–I). Furthermore, plasma TAG concentrations were reduced at 26°C and 30°C at 4, 6 (Supplemental Figure 1, D and E), and 8 weeks posttreatment (Figure 1J). Daily food intake was reduced in mice housed at higher temperatures, as reported previously (Supplemental Figure 1F) (35). These data indicate that FGF21/sTGFBR2 universally blocks diet-induced obesity; however, because circulating TAG is increased at higher environmental temperatures, improvements to hyperlipidemia with FGF21/sTGFBR2 are more striking at thermoneutrality.

### FGF21/sTGFBR2 decreases adiposity, plasma TAG concentrations, and plasma insulin concentrations more effectively at 26°C and 30°C.

**Figure 2:**
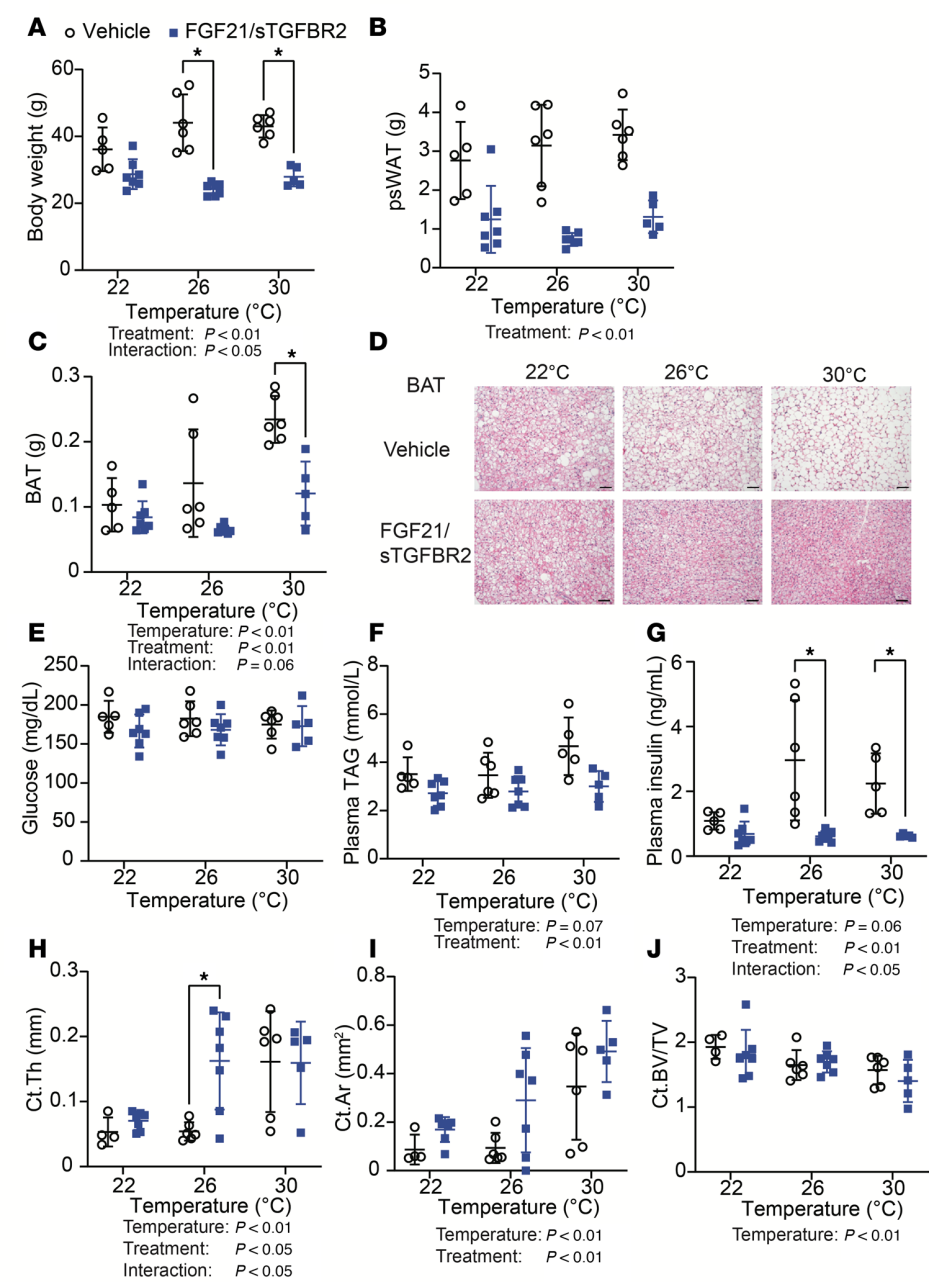
FGF21/sTGFBR2 decreases adiposity and plasma insulin concentrations, with highest efficacy at housing temperatures of 26°C and 30°C. Female mice were euthanized 10 weeks after transduction with FGF21/sTGFBR2 (*n* = 5–7). (**A**) Body weight of mice transduced with FGF21/sTGFBR2 at euthanasia. (**B**) Posterior subcutaneous white adipose tissue (psWAT) and (**C**) brown adipose tissue (BAT) weights. (**D**) Representative histological images of mouse BAT stained with H&E. Scale bar = 50 μm. Fed (**E**) blood glucose concentrations, (**F**) plasma TAG, and (**G**) insulin concentrations prior to euthanasia. Tibial (**H**) cortical thickness (Ct.Th), (**I**) cortical area (Ct.Ar), and (**J**) cortical bone volume fraction (Ct.BV/TV) analyzed via nanoCT. *P < 0.05. Statistical analyses were performed using 2-way ANOVA, followed by Bonferroni’s post hoc test.

Ten weeks of FGF21/sTGFBR2 combination therapy blocked the weight gain observed in vehicle-administered controls, with larger differences observed in mice housed at 26°C or 30°C (Figure 2A). Posterior subcutaneous white adipose tissue (psWAT) weight was decreased in transduced mice across all housing temperatures (Figure 2B). Brown adipose tissue (BAT) weights were also lower in mice treated with FGF21/sTGFBR2, with substantial changes at 30°C, in part because thermoneutrality increased BAT weight in controls (Figure 2C). Due to the decrease in weights of BAT with treatment, we performed histological analyses to investigate potential changes in BAT whitening. BAT was more whitened in mice housed at warmer temperatures, and FGF21/sTGFBR2 restored a BAT-like phenotype with the most improvement at 30°C (Figure 2D). FGF21/sTGFBR2 slightly decreased diaphragm, kidney, and gastrocnemius weights but did not change weights of other organs, including brain, spleen, lungs, and heart (Supplemental Figure 2, A–G). To understand what tissues were most transduced with AAV, we performed a quantitative PCR to recognize a unique AAV DNA sequence in liver, psWAT, and BAT. All tissues were transduced with AAV after treatment, but liver had the highest amount of transduction compared with psWAT and BAT, with increased expression at 30°C (Supplemental Figure 2H). Differences were not observed in fed plasma glucose concentrations (Figure 2E). However, plasma TAG and insulin concentrations were decreased by FGF21/sTGFBR2, particularly at 26°C and 30°C (Figure 2, F and G). Plasma adiponectin concentrations were not changed by treatment but decreased at higher housing temperatures (Supplemental Figure 2, I and J). Tibial cortical bone variables were influenced by housing temperature — thermoneutrality increased cortical thickness, area, and perimeter and decreased cortical bone volume fraction and mineral density (Figure 2, H–J, and Supplemental Figure 3, A and B). FGF21/sTGFBR2 increased cortical thickness (Figure 2H) and perimeter (Supplemental Figure 3A) mostly at 26°C, and cortical area (Figure 2I) was elevated at all temperatures, with the largest induction by FGF21/sTGFBR2 at 26°C. Whereas trabecular bone parameters were mostly unchanged with treatment, trabecular connectivity density was decreased with FGF21/sTGFBR2 at warmer temperatures (Supplemental Figure 3, C–I). Taken together, these data suggest that elevated concentrations of FGF21/sTGFBR2 decrease diet-induced adiposity, improve metabolic dysfunction associated with obesity, and increase certain cortical bone variables, particularly when mice are at warmer environmental temperatures.

### FGF21/sTGFBR2 decreases liver weights and liver TAG concentrations in HFD-fed mice, with greater effects at 26°C and 30°C.

**Figure 3:**
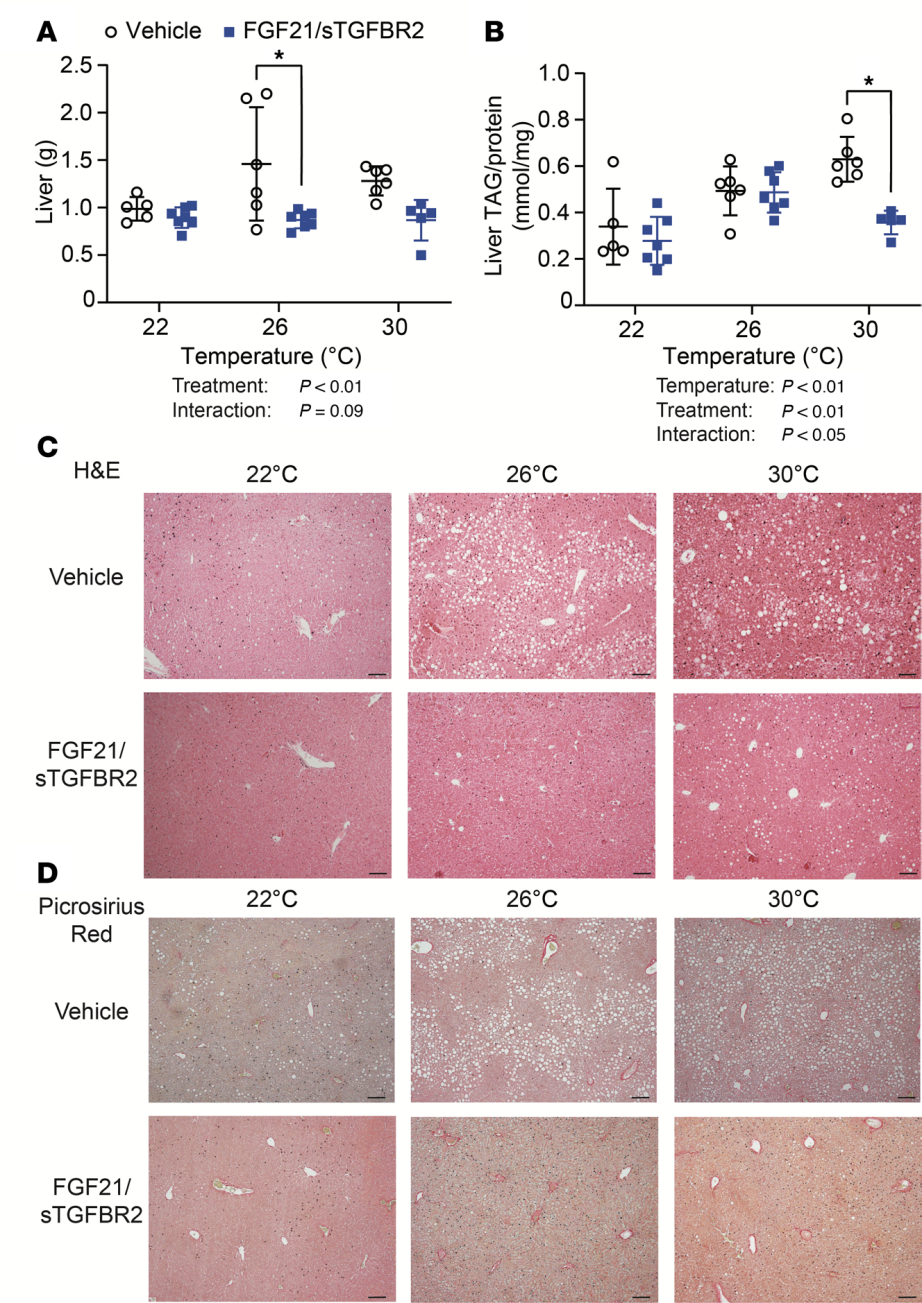
FGF21/sTGFBR2 decreases liver weights and hepatic steatosis, with highest efficacy at housing temperatures of 26°C and 30°C. (**A**) Liver weights in vehicle- and FGF21/sTGFBR2-treated mice at all housing temperatures 10 weeks after transduction (*n* = 6–7). (**B**) Liver TAG concentration normalized to protein amount (*n* = 6–7). (**C**) Representative histological images of mouse livers stained with H&E. Scale bar = 50 μm. (**D**) Representative images of mouse livers stained with Picrosirius red to identify collagen. Scale bar = 50 μm. *P < 0.05. Statistical analyses were performed using 2-way ANOVA, followed by Bonferroni’s post hoc test.

Elevated circulating concentrations of FGF21/sTGFBR2 reduced liver weights, with greatest effects occurring in mice housed at 26°C or 30°C (Figure 3A). Hepatic TAG content increased at warmer temperatures, which made suppression of hepatic lipid by FGF21/sTGFBR2 at 30°C more striking (Figure 3B). Furthermore, histology of liver tissue showed an accumulation of lipid droplets in obese mice, with elevated steatosis observed more at 26°C and 30°C. FGF21/sTGFBR2 treatment partially alleviated effects of HFD, as number and size of hepatic lipid droplets were decreased across all temperatures (Figure 3C). We observed minimal collagen deposition in obese mice as visualized by Picrosirius red — this is likely due to an inadequate length of HFD (36). This collagen deposition was not substantially altered by FGF21/sTGFBR2 (Figure 3D). These data suggest that FGF21/sTGFBR2 treatment is more effective in combating hepatic steatosis in obese mice at higher environmental temperatures.

### FGF21/sTGFBR2 requires adipose tissue to increase insulin sensitivity in HFD-fed mice at thermoneutrality.

**Figure 4:**
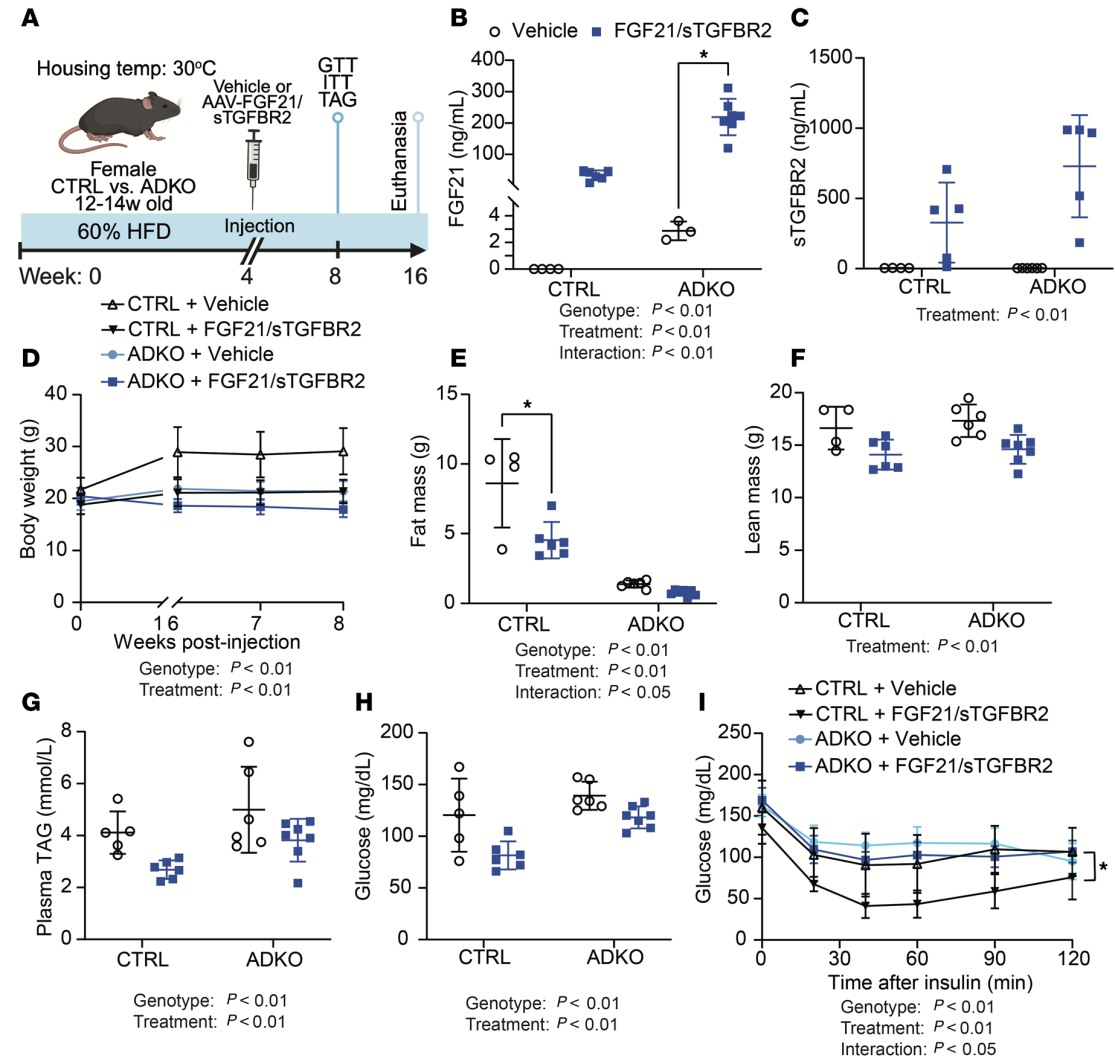
Adipose tissue is required for FGF21/sTGFBR2 to mediate metabolic improvements at thermoneutrality. (**A**) Schematic illustration of housing conditions and experimental timeline; *Lmna*^fl/fl^ = control mice and *Lmna*^ADKO^ = lipodystrophic mice. Plasma (**B**) FGF21 and (**C**) sTGFBR2 concentrations in mice 12 weeks after transduction (*n* = 4–7). (**D**) Body weight in vehicle and FGF21/sTGFBR2 mice 4 weeks after transduction (*n* = 5–7). (**E**) Fat and (**F**) lean mass measured by EchoMRI 4 weeks after transduction (*n* = 4–7). (**G**) Plasma TAG and (**H**) blood glucose concentrations 4 weeks posttreatment (*n* = 5–7). (**I**) Insulin tolerance tests (0.75 U/kg; *n* = 5–7), data presented as mean ± SEM. *P < 0.05. Statistical analyses were performed using 2-way ANOVA, followed by Bonferroni’s post hoc test.

Obesity and lipodystrophy exhibit overlapping metabolic dysfunctions, including insulin resistance and hepatic steatosis (2, 4, 5). To investigate whether metabolic improvements observed in obese mice with FGF21/sTGFBR2 are also observed with lipodystrophy, we studied *Lmna*^ADKO^ lipodystrophic mice in which *Lmna* is knocked out of adipocytes. Our previous findings suggest that FGF21/sTGFBR2 treatment is more effective in obese mice at warmer temperatures; thus, for this experiment we housed female control and lipodystrophic mice at thermoneutrality (30°C). We further challenged their metabolic state by feeding a 60% HFD for 4 weeks. Mice were then administered vehicle or AAVs expressing FGF21 and sTGFBR2 (Figure 4A). At 12 weeks after treatment, concentrations of FGF21 and sTGFBR2 were elevated in control and lipodystrophic mice (Figure 4, B and C). Lipodystrophic mice had an increase in endogenous FGF21 and had a disproportionate increase in plasma FGF21 compared with controls (Figure 4B). Body weight, fat mass, and lean mass were decreased 12 weeks after transduction in both genotypes (Figure 4, D–F). Plasma TAG and glucose concentrations were lowered 4 weeks after treatment in fasted control and lipodystrophic mice (Figure 4, G and H). Whereas FGF21/sTGFBR2 increased insulin sensitivity in controls, this dual therapy did not alter insulin sensitivity in lipodystrophic mice (Figure 4I). These data indicate that though FGF21/sTGFBR2 alters body composition and reduces circulating TAG and glucose similarly in control and lipodystrophic mice, adipose tissue is required for FGF21/sTGFBR2 to improve insulin resistance at thermoneutrality.

### FGF21/sTGFBR2 decreases adiposity and partially improves metabolic dysfunction in control and lipodystrophic mice at thermoneutrality.

**Figure 5:**
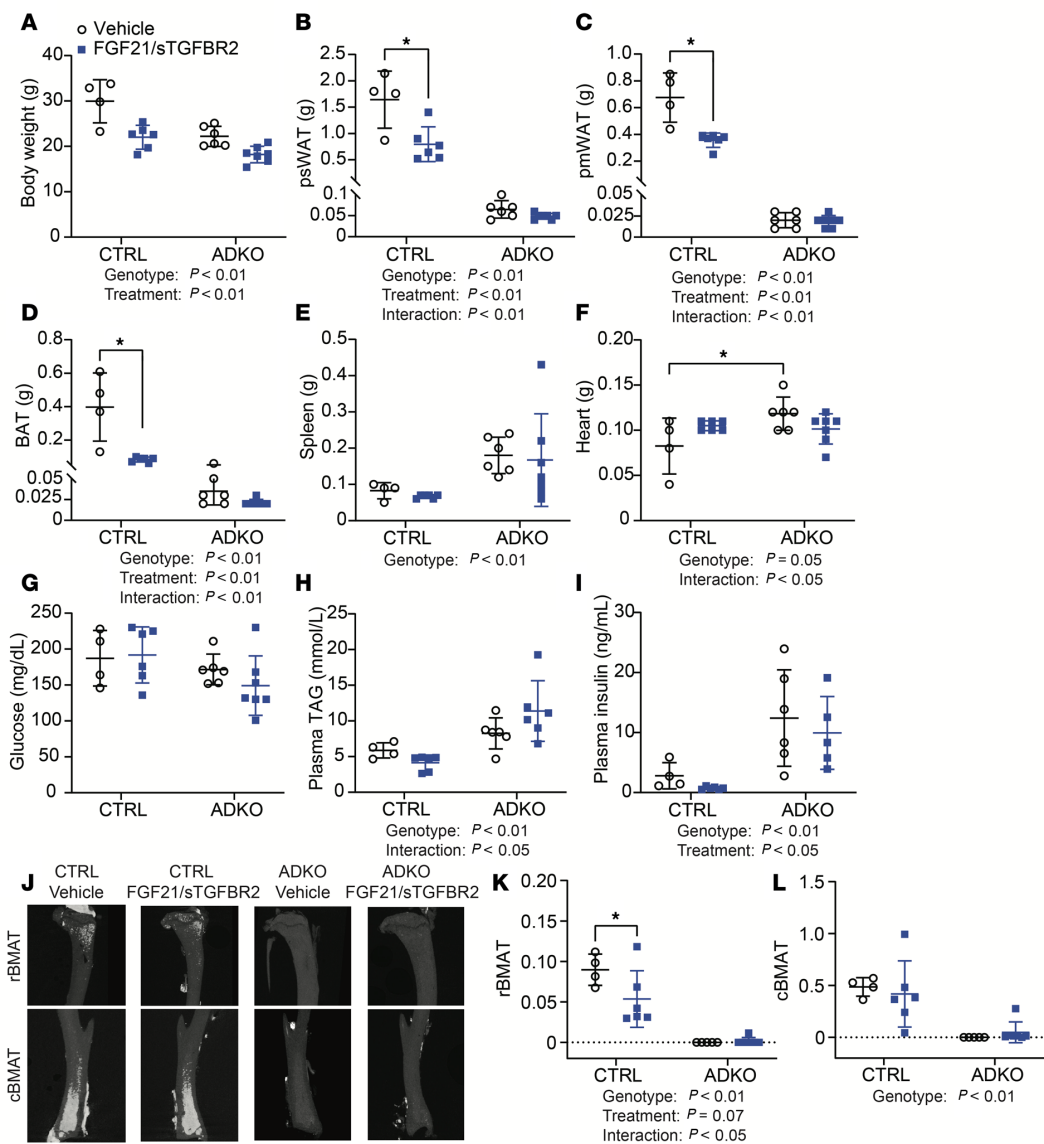
FGF21/sTGFBR2 does not improve metabolic dysfunction of lipodystrophic mice housed at thermoneutrality. Female mice were euthanized 12 weeks after FGF21/sTGFBR2 transduction (*n* = 4–7). (**A**) Body, (**B**) psWAT, (**C**) parametrial white adipose tissue (pmWAT), (**D**) BAT, (**E**) spleen, and (**F**) heart weights in mice at euthanasia. Fed (**G**) blood glucose, (**H**) plasma TAG, and (**I**) plasma insulin concentrations prior to euthanasia. (**J**) Representative tibial osmium-nanoCT images. (**K**) rBMAT normalized to marrow volume. (**L**) cBMAT normalized to marrow volume. *P < 0.05. Statistical analyses were performed using 2-way ANOVA, followed by Bonferroni’s post hoc test.

Mice were euthanized 12 weeks after transduction (Figure 4A), and FGF21/sTGFBR2 reduced body weights in control and *Lmna*^ADKO^ mice (Figure 5A). Adipose tissue weights in control mice decreased after FGF21/sTGFBR2 treatment (Figure 5, B–D). Although lipodystrophic mice had exceedingly low amounts of psWAT, parametrial white adipose tissue (pmWAT), and BAT, adiposity was decreased further by FGF21/sTGFBR2 (Figure 5, B–D). Whereas spleen and heart weights were increased in lipodystrophic mice, FGF21/sTGFBR2 did not alter spleen or heart weights in either genotype (Figure 5, E and F). Kidney weights were decreased after FGF21/sTGFBR2 therapy (Supplemental Figure 4A), but there were no changes in diaphragm or gastrocnemius weights regardless of genotype or treatment. A decrease in brain weight was observed with genotype (Supplemental Figure 4, B–D). Changes were also not observed with treatment or genotype in hematopoiesis at euthanasia as assessed by circulating populations of red blood cells, white blood cell populations, or platelets (Supplemental Figure 5). Differences were also not observed in fed plasma glucose concentrations at euthanasia (Figure 5G). Plasma TAG concentrations were elevated by lipodystrophy, but FGF21/sTGFBR2 did not decrease TAG concentrations in fed lipodystrophic mice 12 weeks posttreatment (Figure 5H). Furthermore, plasma insulin concentrations were also higher in lipodystrophic mice compared with controls in vehicle-treated groups, and FGF21/sTGFBR2 decreased circulating insulin in both genotypes (Figure 5I). Lipodystrophic mice had no detectable circulating adiponectin, and FGF21/sTGFBR2 did not alter adiponectin expression in both genotypes (Supplemental Figure 4, E and F). Similar to the pattern of adiponectin expression, lipodystrophic mice did not have detectable plasma leptin, and the treatment only decreased leptin concentrations in control mice (Supplemental Figure 4G). We also analyzed bone variables and found that FGF21/sTGFBR2 did not affect trabecular or cortical characteristics (Supplemental Figure 6) except for causing a decrease in trabecular connectivity density (Supplemental Figure 6E) and cortical bone volume fraction (Supplemental Figure 6L). We next analyzed regulated and constitutive bone marrow adipose tissues (rBMAT, cBMAT, respectively) (37) and observed that rBMAT in the proximal tibia was depleted in lipodystrophic mice compared with controls (Figure 5, J and K), as observed previously (34). FGF21/sTGFBR2 decreased volume of rBMAT in obese control mice, and the very low amounts of rBMAT in lipodystrophic mice were not altered further by combination therapy (Figure 5, J and K). In obese control mice, cBMAT located in the distal tibia was not altered by FGF21/sTGFBR2 therapy (Figure 5, J and L). In lipodystrophic mice, cBMAT was essentially undetectable by osmium-nanoCT in control and FGF21/sTGFBR2 groups (Figure 5, J and L). In summary, at thermoneutrality FGF21/sTGFBR2 more effectively improves metabolic parameters in HFD-fed obese mice than in HFD-fed mice with lipodystrophy, and FGF21/sTGFBR2 decreases quantity of rBMAT only in mice with diet-induced obesity.

### FGF21/sTGFBR2 treatment reduces liver weight, total hepatic TAG amount, and liver collagen deposition in lipodystrophic mice housed at 30°C.

**Figure 6:**
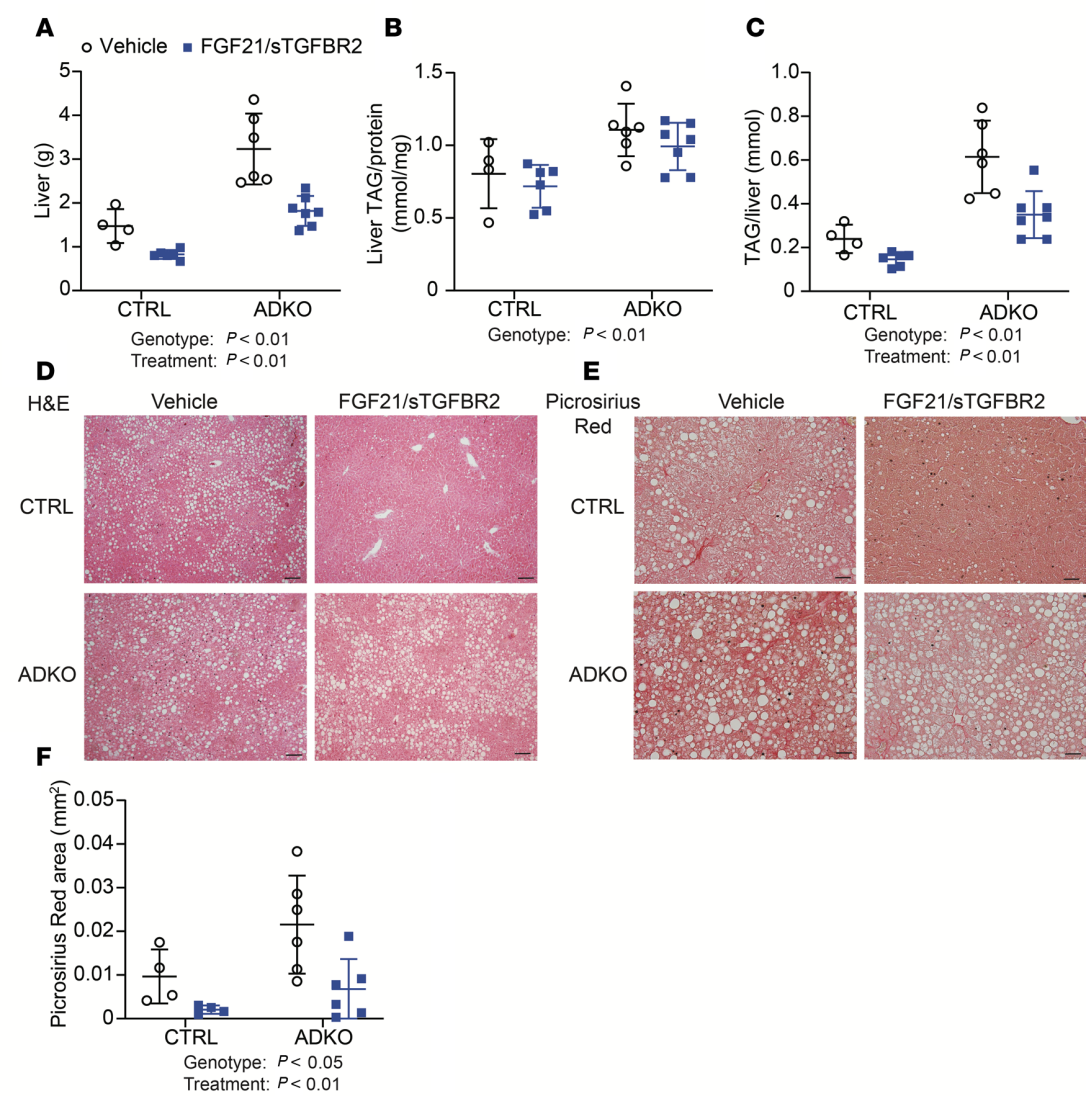
FGF21/sTGFBR2 reduces liver weight, total hepatic TAG, and liver collagen deposition in lipodystrophic mice housed at 30°C. Female mice were euthanized 12 weeks after FGF21/sTGFBR2 transduction (*n* = 4–7). (**A**) Liver weights at euthanasia. (**B**) Liver TAG normalized to protein content. (**C**) TAG amount normalized to total liver weight. (**D**) Representative histological images of mouse livers stained with H&E. Scale bar = 50 μm. (**E**) Representative images of mouse livers stained with Picrosirius red for collagen. Scale bar = 50 μm. (**F**) Machine learning quantification of area stained by Picrosirius red in the liver. Statistical analyses were performed using 2-way ANOVA, followed by Bonferroni’s post hoc test.

*Lmna*^ADKO^ mice had greater liver weights than control mice regardless of treatment, but FGF21/sTGFBR2 reduced liver weights in both control and *Lmna*^ADKO^ mice (Figure 6A). Hepatic TAG content was significantly elevated in lipodystrophic mice relative to controls (Figure 6B). However, transduction with FGF21/sTGFBR2 did not reduce TAG amount normalized to protein content in either genotype (Figure 6B). Notably, a reduction in total hepatic TAG content after normalization to liver weight was observed in control and lipodystrophic mice at 30°C (Figure 6C). Histology of liver tissue showed a decrease in the number of lipid droplets with FGF21/sTGFBR2 treatment in control mice but not in lipodystrophic mice (Figure 6D). Furthermore, liver collagen deposition, visualized by Picrosirius red staining, was higher in lipodystrophic mice, and this hepatic fibrosis was visually and quantitatively reduced following transduction with FGF21/sTGFBR2 in both genotypes (Figure 6, E and F). In summary, FGF21/sTGFBR2 reduced liver weights, hepatic TAG amount, and liver fibrosis in lipodystrophic mice housed at 30°C.

### FGF21 improves insulin sensitivity of lipodystrophic mice housed at 22°C.

**Figure 7:**
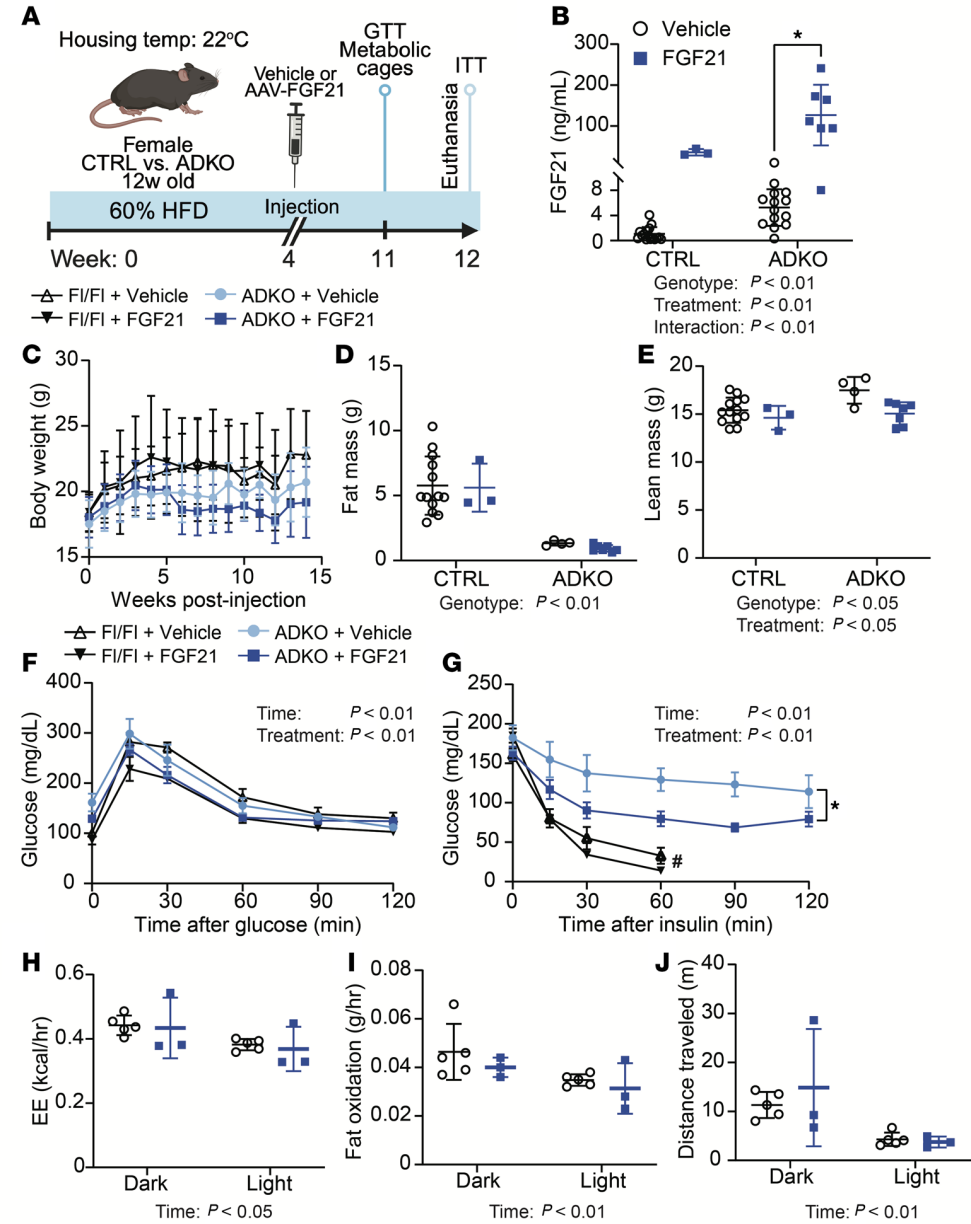
FGF21 improves insulin sensitivity of lipodystrophic mice housed at 22°C. (**A**) Schematic illustration of housing conditions and experimental timeline. (**B**) Plasma FGF21 concentrations 8 weeks post-transduction (*n* = 3–16). (**C**) Body weight, (**D**) fat mass, and (**E**) lean mass measured by EchoMRI 7 weeks posttransduction (*n* = 3–13). (**F**) Glucose tolerance test (1 mg/kg; *n* = 3–13), data presented as mean ± SEM. (**G**) Insulin tolerance test (0.75 U/kg; *n* = 3–13), data presented as mean ± SEM. #Mice in **G** received intraperitoneal glucose because their blood glucose dropped below 30 mg/dL. Indirect calorimetry was measured for 3 days using the Promethion system. (**H**) Energy expenditure (EE) averaged during dark or light cycles, (**I**) fat oxidation averaged during the first 4 hours of dark or light cycles, and (**J**) distance traveled averaged during dark or light cycles. *P < 0.05. Statistical analyses were performed using 2-way ANOVA, followed by Bonferroni’s post hoc test.

Previous studies have tested multiple therapeutic combinations with FGF21 or FGF21 alone and have found that FGF21 alone is sufficient to improve insulin sensitivity and reduce obesity in HFD-fed mice (23). We were particularly interested in effects of FGF21 alone because multiple studies reported that FGF21 alone as a gene therapy slows aging-related functional decline and improves metabolic dysfunction and is safe in larger animals, including dogs and nonhuman primates (22, 38, 39). In lipodystrophic mice housed at 30°C, FGF21/sTGFBR2 decreased body weight and liver TAG but did not improve insulin sensitivity. To determine if insulin sensitivity of *Lmna*^ADKO^ mice is influenced by FGF21 treatment at standard environmental temperature, we housed control and lipodystrophic mice at 22°C and fed them an HFD for 4 weeks before FGF21 administration (Figure 7A). Similar to our previous findings at 30°C, lipodystrophic *Lmna*^ADKO^ mice had higher circulating concentrations of endogenous FGF21 at 22°C and a disproportionate increase in plasma FGF21 after transduction compared with controls (Figure 7B). Whereas lipodystrophic mice had reduced body weight and fat mass at 22°C, elevated concentrations of FGF21 did not decrease body weight or fat mass further in these mice (Figure 7, C and D). However, lean mass was decreased in control and lipodystrophic mice after FGF21 administration (Figure 7E). Glucose and insulin tolerance tests conducted 7 to 8 weeks after transduction with FGF21 revealed improved glucose and insulin sensitivity in control and *Lmna*^ADKO^ mice at 22°C (Figure 7, F and G). To investigate whether FGF21 influences energy expenditure in lipodystrophic mice, we performed indirect calorimetry on a subset of *Lmna*^ADKO^ mice. FGF21 treatment in lipodystrophic mice did not alter energy expenditure, fat oxidation, and distance traveled during light and dark cycles (Figure 7, H–J). These data suggest that adipose tissue is not required for FGF21 to improve insulin sensitivity in mice housed at 22°C.

### FGF21 does not improve hyperlipidemia or hyperinsulinemia of lipodystrophic mice housed at 22°C.

**Figure 8:**
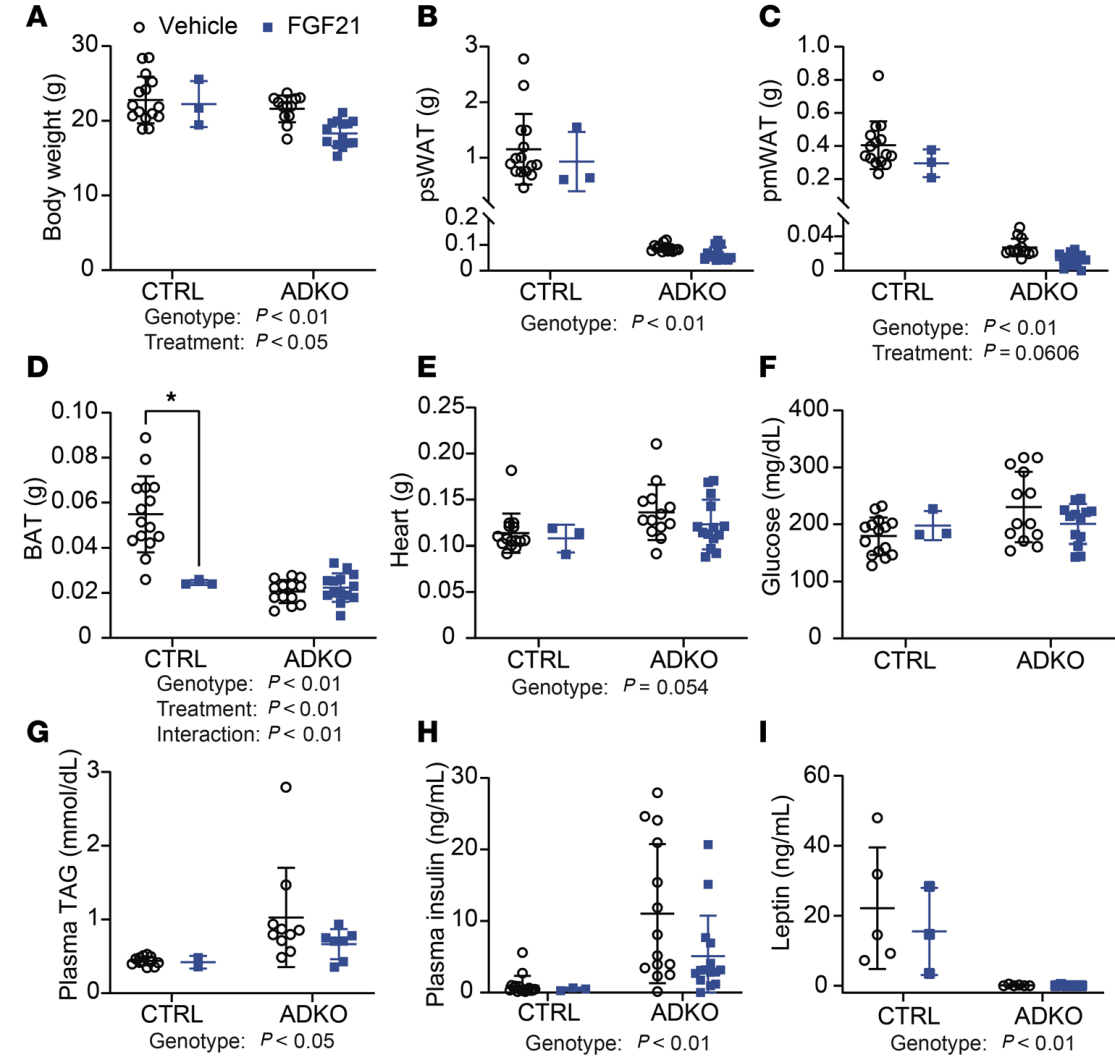
FGF21 does not improve metabolic dysfunction of lipodystrophic mice housed at 22°C. Female mice were euthanized 8 weeks after FGF21 administration (*n* = 3–15). (**A**) Body, (**B**) psWAT, (**C**) pmWAT (**D**) BAT, and (**E**) heart weights at euthanasia. Fed (**F**) blood glucose, (**G**) plasma TAG, (**H**) plasma insulin, and (**I**) plasma leptin concentrations prior to euthanasia. *P < 0.05. Statistical analyses were performed using 2-way ANOVA, followed by Bonferroni’s post hoc test.

Mice from Figure 7 were analyzed after euthanasia. Eight weeks after FGF21 transduction at 22°C reduced body weights but not weights of psWAT, pmWAT, retroperitoneal WAT (rpWAT), or perirenal WAT (prWAT) in control or lipodystrophic mice (Figure 8, A–C, and Supplemental Figure 7, A and B). As expected, all these variables including BAT were reduced in *Lmna*^ADKO^ mice regardless of treatment. Although FGF21 reduced BAT weights in controls, treatment had little effect on BAT in lipodystrophic mice (Figure 8D). Although lipodystrophic mice had greater spleen and heart weights and smaller tibia lengths than controls, there were no changes in weights of heart, kidney, diaphragm, lungs, brain, spleen, or tibia lengths after FGF21 treatment regardless of genotype (Figure 8E and Supplemental Figure 7, C–H). Circulating TAG and insulin concentrations were elevated in *Lmna*^ADKO^ mice, but FGF21 did not alter concentrations of glucose, TAG, or insulin in control or *Lmna*^ADKO^ mice at 22°C (Figure 8, F–H). Both adiponectin and leptin were undetectable in lipodystrophic mice, and the treatment did not affect amounts of either adipokine regardless of genotype (Figure 8I and Supplemental Figure 7, I and J). Taken together, these data suggest that FGF21 does not alter adiposity or improve metabolic dysfunction of lipodystrophic mice housed at 22°C.

### FGF21 treatment alone reduces liver weight, total hepatic TAG, and liver collagen deposition in lipodystrophic mice housed at 22°C.

**Figure 9:**
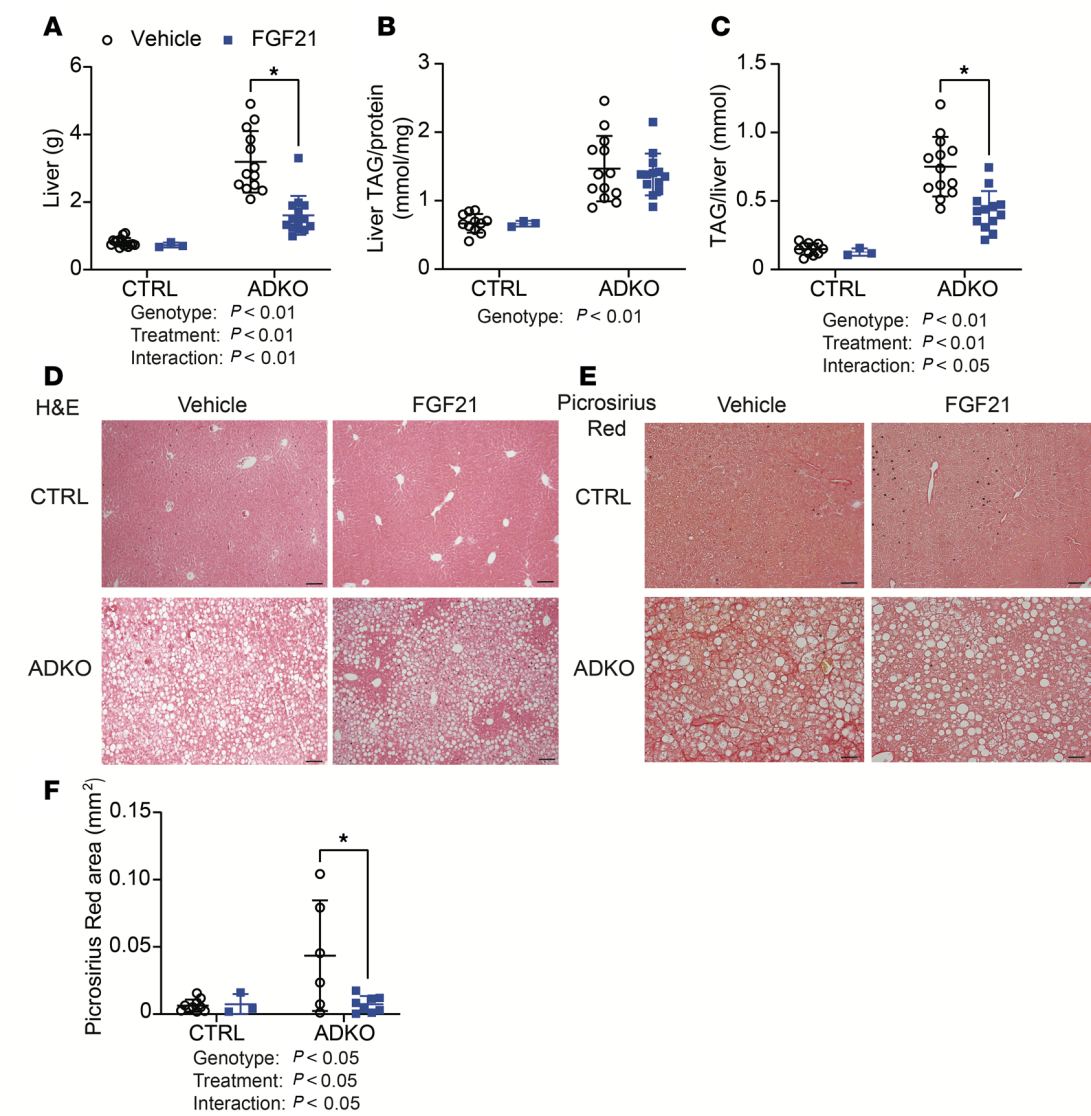
FGF21 reduces liver weight, total hepatic TAG content, and liver collagen deposition in lipodystrophic mice housed at 22°C. Female mice were euthanized 8 weeks after FGF21 administration (*n* = 3–15). (**A**) Liver weights at euthanasia. (**B**) Liver TAG normalized to protein content. (**C**) Total liver TAG normalized to liver weights. (**D**) Representative histological images of H&E-stained mouse livers. Scale bar = 50 μm. (**E**) Representative images of mouse livers stained with Picrosirius red for collagen. Scale bar = 50 μm. (**F**) Machine learning quantification of area stained by Picrosirius red in the liver. *P < 0.05. Statistical analyses were performed using 2-way ANOVA, followed by Bonferroni’s post hoc test.

*Lmna*^ADKO^ mice had greater liver weights than control mice, and FGF21 administration reduced liver weights in control and *Lmna*^ADKO^ mice when housed at 22°C (Figure 9A). Hepatic TAG content was higher in lipodystrophic mice than controls, but it was not reduced by FGF21 transduction (Figure 9B). Similar to our experiment at 30°C, a decrease in total hepatic TAG was observed in lipodystrophic mice at 22°C when normalized to total liver weight (Figure 9C). Histology of liver tissue showed that number and size of lipid droplets were substantially greater in lipodystrophic mice compared with controls (Figure 9D). However, FGF21 did not visually reduce lipid droplet accumulation in controls and lipodystrophic mice (Figure 9D). Liver collagen stained by Picrosirius red was higher in lipodystrophic mice than control mice at 22°C, and this hepatic fibrosis was reduced visually and quantitatively by FGF21 treatment (Figure 9, E and F). These data suggest that, as observed in Figure 6 for dual treatment at 30°C, FGF21 administration reduces liver weights, hepatic TAG amount, and collagen deposition in lipodystrophic mice housed at 22°C.

### Bulk RNA-Seq analyses of livers reveal that FGF21 decreases hepatic cell proliferation and fibrosis in lipodystrophic mice housed at 22°C.

**Figure 10:**
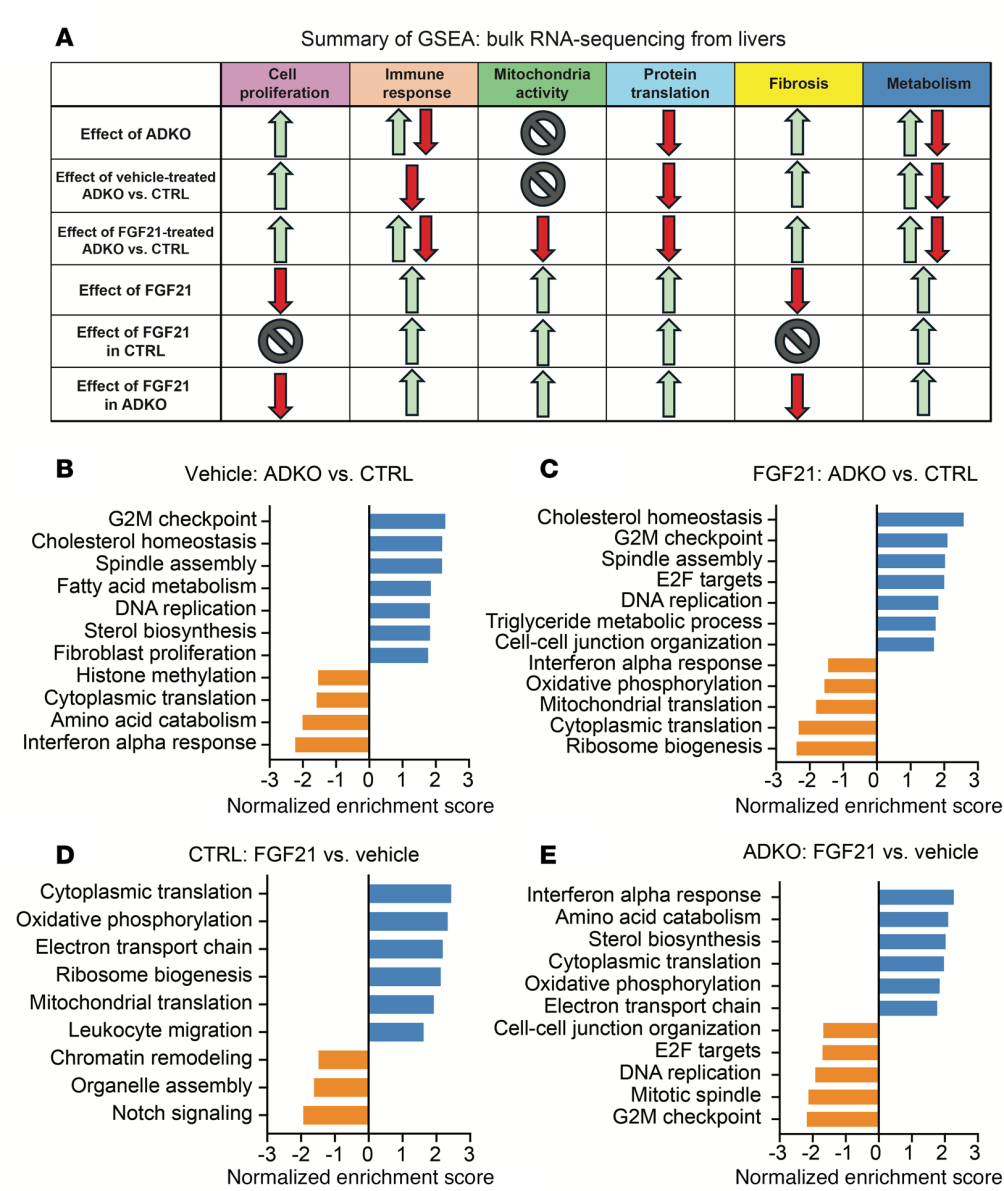
RNA-Seq analyses of livers reveal that livers from lipodystrophic mice have increased cell proliferation and increased fibrosis and that FGF21 treatment increases mitochondrial and translation pathways. (**A**) Summary of key GSEA pathways in hepatic mRNA of CTRL versus ADKO mice 8 weeks after treatment with FGF21 or vehicle. (**B**) GSEA pathways changed in CTRL mice dependent on treatment, padj < 0.05. (**C**) GSEA pathways changed in ADKO mice dependent on treatment, padj < 0.05. (**D**) GSEA pathways changed in vehicle-treated mice dependent on genotype, padj < 0.05. (**E**) GSEA pathways changed in FGF21-treated mice dependent on genotype, padj < 0.05.

To investigate hepatic molecular changes that occur with lipodystrophy and FGF21 treatment, bulk RNA-Seq analyses were performed, and data were analyzed using gene set enrichment analysis (GSEA) to identify transcriptomic patterns (Figure 10A). First, we were interested in how lipodystrophy affects the hepatic transcriptome. Lipodystrophic mouse livers had upregulation of cell proliferation and fibrosis, with increased regulation of the G2M checkpoint, spindle assembly, DNA replication, and fibroblast proliferation (Figure 10, B and C). In addition to increased hepatic steatosis, this signature may partially explain the large increase in liver weights at baseline in lipodystrophic mice. Furthermore, cholesterol homeostasis and sterol biosynthesis were increased, supporting the observed increased hepatic steatosis. Immune response was mixed in lipodystrophic mice compared with controls when treated with vehicle: interferon-α and -β responses were downregulated, but macrophage migration and TNF signaling were increased. Protein translation was also downregulated in lipodystrophic mice regardless of treatment.

Next, the impact of FGF21 was examined. FGF21 transduction led to an upregulation of immune response pathways, such as increased leukocyte migration in control mice and enhanced interferon-α and -γ responses in *Lmna*^ADKO^ mice (Figure 10, D and E). Mitochondrial activity was also upregulated in both genotypes after FGF21 treatment, as evidenced by increased expression in gene sets related to oxidative phosphorylation, the electron transport chain, and mitochondrial translation (Figure 10, D and E). Additionally, FGF21 promoted protein translation and metabolic processes in livers of control and lipodystrophic mice, including amino acid metabolism and sterol biosynthesis (Figure 10, D and E). Several pathways were downregulated following FGF21 transduction in control mice, including Notch signaling and organelle assembly (Figure 10D). In contrast, in lipodystrophic mice, FGF21 transduction led to downregulation of cell proliferation and fibrosis, with a decrease in the regulation of G2M checkpoint, mitotic spindle, DNA replication, and E2F target pathways (Figure 10E). In summary, FGF21 treatment increases hepatic immune response, mitochondrial activity, and lipid metabolism across genotypes. The reduction by FGF21 of liver weights and fibrosis in lipodystrophic mice can be explained, in part, by decreased cell proliferation and fibrosis pathways.

## Discussion

Given the unmet clinical need for patients with *LMNA*-induced lipodystrophy, we designed this study to explore the impact of a gene treatment using viral mediated delivery of FGF21 and sTGFBR2 in a preclinical model of *LMNA*-induced lipodystrophy created in our lab. To understand the impact of this therapy, we simultaneously treated obese mice. Both the obesity and lipodystrophy groups were housed at different environmental temperatures to ensure that metabolic dysfunction was uncovered in both disease states. First, we found that in HFD-induced obesity, warmer environmental housing temperatures improved effectiveness of FGF21/sTGFBR2 in decreasing body weight, improving insulin resistance, reducing plasma TAG, and decreasing hepatic steatosis. Part of this greater efficacy at warmer housing temperatures was due to elevated baseline metabolic dysfunction in mice housed at 26°C and 30°C, as reflected in body weight, circulating TAG, and liver TAG in vehicle-treated mice. This finding is consistent with previous literature showing decreased energy expenditure and increased weight gain, glucose intolerance, and hyperlipidemia in mice housed at 30°C (27, 40, 41). FGF21 administration to HFD-induced obese mice caused similar decreases in weight loss at 22°C and 30°C (31). This study (31) differed in design from ours in that we used female mice rather than male, and we used an AAV to continuously deliver mouse FGF21/sTGFBR2; they used human FGF21 administered twice daily subcutaneously for 3 weeks. The previous study (31) did find that FGF21-treated mice housed at 22°C had higher energy expenditure than mice housed at 30°C, which we did not explore in our study. Although prior work suggested effects of human FGF21 on mice were independent of environmental temperature (31), our results suggest that with FGF21/sTGFBR2 administration to obese mice, housing temperatures of 26°C or 30°C may be better than 22°C to detect metabolic effects and to mimic human thermoneutral conditions.

We also characterized on HFD-fed lipodystrophic mice the effects of FGF21/sTGFBR2 combination therapy at 30°C or FGF21 alone at 22°C. Gene therapy did not restore normal adipose tissue function in lipodystrophic mice as measured by adiponectin and leptin secretion in circulation, perhaps due to the exceedingly low remaining adipocyte number. We observed that FGF21/sTGFBR2 treatment in control and lipodystrophic *Lmna*^ADKO^ mice housed at thermoneutrality reduced circulating TAG and glucose 4 weeks posttransduction but did not improve insulin resistance in lipodystrophic mice. However, FGF21 administration alone at 22°C did improve insulin resistance in *Lmna*^ADKO^ mice, but not other metabolic parameters, suggesting that FGF21 potentially interacts with nonshivering thermogenesis mechanisms to improve insulin sensitivity in these mice (42). The interplay between temperature and BAT may explain why metabolic improvements are observed under certain conditions. In our studies of obese mice, we saw that FGF21/sTGFBR2 treatment restored a BAT-like phenotype at warmer temperatures, which is a likely contributor to the observed metabolic improvements. However, lipodystrophic mice have reduced BAT, and we only see an improvement in metabolism in treated lipodystrophic mice housed at 22°C, highlighting the possibility of functional BAT or thermogenic WAT in lipodystrophic mice housed at 22°C versus 30°C. Support for this idea comes from investigation of global *Bscl2*-KO (seipin) mice. In this model of lipodystrophy, an FGF21 analog, LY2405319, improved insulin sensitivity in young mice housed at 22°C (25). In contrast, but similar to our findings at 30°C, adipose tissue was required for FGF21 to reduce circulating insulin concentrations in aP2-nSREBP1c lipodystrophic mice fed a normal chow diet, which was proposed to result from impaired FGF21 signaling in lipodystrophic adipose tissue (24). This study differed from ours in a number of respects. In addition to the genetic modification causing the lipodystrophy, *nSREBP1c* overexpression versus *Lmna* deficiency, aP2 (i.e., *Fabp4*) drives *Cre* expression more broadly than *Adipoq*, including in adipose tissue macrophages (43), providing one possible reason that the phenotype of aP2-nSREBP1c mice is more extreme than *Lmna*^ADKO^ mice (44). Expression of nuclear SREBP1c from the aP2 locus caused perinatal distension of the abdomen, interscapular humps, and impaired growth, with some pups dying prior to weaning. As adults, the mice had severe hepatic steatosis and hyperinsulinemia (44). Another factor that may have influenced differences between studies is background mouse strain. aP2-nSREBP1c mice are on an FVB background (45), which have about 60 genetic variants that directly affect protein structure or mRNA stability, and provide resistance to diet-induced obesity, whereas *Lmna*^ADKO^ mice are on a C57BL/6J background, which also has genetic variants that affect protein function but that increase susceptibility to diet-induced obesity (46, 47). These studies suggest in addition to environmental temperature, other variables, including the genetic basis for lipodystrophy, background strain, and diet, may influence metabolic benefits of FGF21.

The necessity for functional adipose tissue to mediate effects of FGF21 is also illustrated by observations that insulin sensitivity did not improve after FGF21 administration in HFD-induced obese mice with an adipocyte-specific knockout of the coreceptor for FGF signaling, β-Klotho (48). This raises the possibility that *Lmna*^ADKO^ lipodystrophic mice, when housed at 30°C, could have a defect in FGF21 signaling in adipose tissue, which may explain the increased insulin sensitivity. FGF21 signaling in nonadipose organs may also contribute to improvements in insulin sensitivity in lipodystrophic mice at 22°C, especially signaling in liver and muscle. Consistent with this notion, expression of *Fgfr2* and *Fgfr4* is decreased in vehicle-treated *Lmna*^ADKO^ livers relative to controls, suggestive of an FGF21 signaling defect. Our study does differ from previous studies of FGF21 in lipodystrophy in that 1) AAV gene therapy was used to administer FGF21, 2) our model of lipodystrophy used an *Adipoq-*Cre driver to knock out *Lmna*, which is expressed specifically in adipocytes and marrow adipogenic lineage progenitors (49), 3) *Lmna*^ADKO^ mice were on a C57BL/6J background whereas aP2-nSREBP1c were on an FVB background, 4) *Lmna*^ADKO^ mice were on an HFD, and 5) we investigated effects at 22°C and 30°C housing temperatures. Taken together, in our model of *Lmna*^ADKO^ lipodystrophy, adipose tissue is required to mediate metabolic improvements after FGF21 administration at housing temperatures of 30°C, but not 22°C, which adds to the complex and at times contradictory literature on metabolic improvements with FGF21 treatment of preclinical models of lipodystrophy.

We observed widespread transcriptomic changes in response to lipodystrophy in this study, with 1,953 differentially expressed genes between vehicle-treated lipodystrophic and control livers and 888 genes that differed between FGF21-treated lipodystrophic and control livers. Livers from lipodystrophic mice had increased GSEA pathway expression related to cell proliferation and fibrosis, and decreased protein translation, and multiple changes in opposing directions related to immune response and metabolism. To our knowledge, this is the first RNA-Seq dataset to profile differences in the liver transcriptome in a mouse model of partial lipodystrophy, in which effects on the liver are likely secondary to metabolic dysfunction resulting from loss of adipose tissue. Interestingly, FGF21 had minor effects on hepatic gene expression, with 42 differentially expressed genes between FGF21 and vehicle control livers, and only 5 genes differentially expressed between FGF21 and vehicle lipodystrophic livers. When analyzed with GSEA, FGF21 treatment increased sterol biosynthesis, cholesterol homeostasis, and triacylglycerol metabolism pathways, which was similar to previously published hepatic RNA-Seq of acute FGF21 treatment in diet-induced obese mice (50). Transcriptomic analyses of adipose tissue after FGF21 treatment in nonhuman primates also had similar changes to our hepatic RNA-Seq dataset, including increased oxidative phosphorylation, mitochondria, and lipid synthesis pathways (38). FGF21 is known to induce hepatic expression of PGC1α and its downstream genes, which aligns with our observed increase in mitochondrial gene expression (51). Future investigations of how FGF21 alters fibrosis, protein translation, and cell proliferation in lipodystrophic and control livers could explain the observed decrease in liver size but not hepatic steatosis.

The effects of FGF21 on bone mass and BMAT have been controversial. Early reports suggested that elevated FGF21 as a result of transgenesis, AAV delivery, or injection of recombinant protein in male mice all resulted in loss of bone mass and bone mineral density and increased BMAT, with elevated bone and decreased BMAT observed in *Fgf21*-KO mice (52, 53). Subsequent investigation, however, found that in male diet-induced obese mice, human FGF21 administration did not affect bone loss or BMAT, similar to our results with FGF21/sTGFBR2 combination therapy for trabecular and cortical bone in HFD-fed mice at 22°C (54). Human FGF21 also did not change bone mass in obese nonhuman primates (55). An aging study from the Scherer lab with overexpression of FGF21 from adipocytes found that 1.7 years of elevated circulating FGF21 did not influence bone characteristics, such as tibial length or trabecular and cortical bone mineral density (39).

In our experiments, FGF21/sTGFBR2 administration increased cortical area and thickness only at warmer housing temperatures. We also observed a depletion of tibial rBMAT and cBMAT in HFD-fed *Lmna*^ADKO^ mice housed at 30°C but did not see changes in bone mass, contrary to the mild increase in cortical bone observed previously in female lipodystrophic *Lmna*^ADKO^ mice at 22°C (34). In contrast, loss of marrow adiposity due to expression of diphtheria antigen increased trabecular and cortical bone mass in both sexes (56). Taken together, these data suggest that the mechanism of BMAT loss, in addition to diet and housing temperature, greatly influences whether bone mass is altered. Furthermore, we found that in female diet-induced obese mice housed at 30°C, FGF21/sTGFBR2 treatment decreased the amount of tibial rBMAT, contrary with previous findings that showed no change in femoral rBMAT with FGF21 treatment in male diet-induced obese mice at 22°C (54). Therefore, effects of FGF21 treatment on BMAT are at least partially dependent on housing temperature of mice and could be further confounded by rBMAT location, sex of mice, and HFD duration.

We administered lipodystrophic mice with a dual FGF21/sTGFBR2 treatment or with FGF21 alone. Differential improvements in insulin sensitivity and lipid metabolism were likely due to the 2 housing temperatures evaluated, since room temperature (22°C) versus thermoneutrality (30°C) is known to have a profound effect on the transcriptome of adipose and other tissues (57). FGF21 likely mediates most of the metabolic effects observed due to its known role in liver, adipose, and metabolic homeostasis; FGF21 alone or dual FGF21/sTGFBR2 causes the same degree of weight loss in obese mice (23). However, the added effect of sTGFBR2 cannot be ignored, especially in targeting liver fibrosis in lipodystrophy and in stopping progression of heart failure (23). Future investigations are required to confirm whether sTGFBR2 contributes substantially to improvements to metabolic dysfunction and hepatic steatosis in lipodystrophic mice at different housing temperatures. Another group used a dual gene therapy of human adiponectin and leptin in *ob/ob* mice, which improved metabolic dysfunction, highlighting the potential of this therapeutic approach (58). Furthermore, the McIlroy and Rochford groups have restored human BSCL2 expression in global *Bscl2*-KO mice via AAV, which improved lipodystrophy-associated metabolic issues, an exciting application of gene therapy in lipodystrophy (59, 60).

A limitation of our study is that we only used female mice in our models of obesity and lipodystrophy. It has been shown that effects of FGF21 on energy balance, liver TAG, signaling in WAT, and adiponectin secretion in diet-induced obese mice are sex dependent (50). Thus, future studies are necessary to determine whether FGF21 alone or dual FGF21/sTGFBR2 have different metabolic improvement effects in lipodystrophic male mice. Another limitation is that the utilized lipodystrophic mouse model is a genetic knockout model rather than a missense variant model, which is the genetic basis of disease in patients with lipodystrophy. In conclusion, this study demonstrated that 1) FGF21/sTGFBR2 dual treatment improves metabolic dysfunction more at warmer housing temperatures, 2) FGF21/sTGFBR2 slightly improves metabolic dysfunction in lipodystrophic mice housed at 30°C but not insulin resistance, and 3) FGF21 alone improves metabolic dysfunction in lipodystrophic *Lmna*^ADKO^ mice housed at 22°C and decreases liver weight and fibrosis through effects on the hepatic transcriptome. This investigation contributes to our understanding of gene therapy for obesity and lipodystrophy, and it highlights the importance of environmental housing temperature in determining treatment efficacy in preclinical models of metabolic disease.

## Methods

### Sex as a biological variable.

All mice used in this study were females. Lipodystrophy is more commonly diagnosed in females, and the lipodystrophic mice used in this study have a more severe metabolic phenotype in females (34). Other mouse models of lipodystrophy also have a sexual dimorphic effect on metabolic dysfunction, with both AZIP mice and global *Bscl2*-KO female mice having worsened metabolic parameters compared with males (61, 62). It is unknown whether lipodystrophic male mice would have the same effects of FGF21 or FGF21/sTGFBR2 treatment at different environmental temperatures, which is a limitation of this study and should be evaluated in the future. It has been previously shown that obese male mice benefit metabolically from FGF21/sTGFBR2 administration at a housing temperature of 22°C (23).

### Animals.

C57BL/6J (000664) and *Adipoq*-Cre mice (028020) mice were from The Jackson Laboratory. *Lmna*^fl/fl^ mice had *loxP* sites flanking exons 10 and 11 of the *Lmna* allele (63). Animals described as controls were *Lmna*^fl/fl^ *Adipoq-*Cre^–^, and *Lmna*^ADKO^ were *Lmna*^fl/fl^ *Adipoq*-Cre*^+^*. Control and *Lmna*^ADKO^ mice were 12–14 weeks old at the start of experiments. C57BL/6J mice were 8 weeks old at the start of experiments. Female mice were analyzed throughout these studies. Mice were euthanized by inhaled isoflurane overdose, death was assured via cervical dislocation or bilateral pneumothorax, and adipose depots were dissected as previously described (64). Animals were group-housed at 22°C, 26°C, and 30°C at a relative humidity of 30%–50% with a 12-hour light/12-hour dark cycle and free access to water and food. Mice were fed ad libitum 60% HFD (D12492, Research Diets), and food was changed twice per week. Temperature sensors were implanted intraperitoneally, and core body temperatures were measured with an external scanner. Fat and lean mass was measured in live animals with an EchoMRI-100H (EchoMRI LLC). Daily care of mice was overseen by the Unit for Laboratory Animal Medicine at the University of Michigan.

### AAV administration.

AAV8 vectors were synthesized as previously described, with mouse versions of FGF21 and sTGFBR2 expressed (23). AAV-FGF21/sTGFBR2 or vehicle (PBS pH 7.4 + 0.001% F-68 Pluronic) was delivered via retro-orbital injections. In the temperature efficacy experiments, mice were dosed at 5 × 10^11^ viral genomes per mouse in 50 μL; in the lipodystrophy experiments, mice were dosed at the same concentration but in 100 μL to reduce technical error. All mice were randomized based on weight for each experiment.

### Glucose and insulin tolerance tests.

For glucose tolerance tests, mice were fasted overnight (12–16 hours) and administered an intraperitoneal injection of glucose in water (1 mg/kg body weight). For insulin tolerance tests, mice were fasted 4 hours and administered an intraperitoneal injection of insulin in saline (0.75 U insulin/kg body weight; Eli Lilly and Company). Blood was collected from the tail vein, and glucose concentrations were monitored using a glucometer and Contour Next blood glucose strips (Bayer AG).

### Serum measurements.

Blood was collected from tail vein or at euthanasia via inferior vena cava and was allowed to coagulate on ice for 2 hours. Mice were fasted at bleeds for 4 hours unless otherwise stated in the figure legend. After centrifugation at 2,000*g* for 20 minutes at 4°C, serum was collected and stored at –80°C. ELISA was used to measure circulating concentrations of leptin (900-K76K, Invitrogen), insulin (90080, Crystal Chem), murine FGF21 (MF2100, R&D Systems), and murine sTGFBR2 (ab277719, Abcam). TAG was measured via colorimetric assay (10010303, Cayman Chemical). Serum adiponectin was measured via immunoblot (adiponectin antibody: Sigma-Aldrich A6354; albumin: Thermo Fisher Scientific PA5-27707).

### Complete blood count.

Blood was collected at euthanasia and stored in EDTA-coated tubes. Blood was analyzed by University of Michigan’s Unit for Laboratory Animal Medicine Pathology Core using a Heska Element HT5 Veterinary Hematology Analyzer.

### Liver TAG.

Liver TAG was measured via colorimetric assay according to manufacturer protocol (10010303, Cayman Chemical). Briefly, liver pieces were weighed and minced into small pieces on ice. Minced tissue was homogenized in 2 mL of NP-40 substitute assay reagent containing protease inhibitors, then centrifuged at 10,000*g* for 10 minutes at 4°C, and supernatant was transferred to another tube. Samples were then loaded into a 96-well plate along with TAG standards and an enzyme mixture to result in a coupled enzymatic reaction system to measure glycerol, which was measured at 530–550 nm using a plate reader. For normalization, protein concentrations were determined from liver supernatants via bicinchoninic acid assay (23225, Thermo Fisher Scientific).

### Mouse liver and BAT histology.

After collection, tissues were fixed for 72 hours in 10% neutral-buffered formalin at 4°C. Tissues were rinsed with PBS and dehydrated in a graded series of ethanol washes, processed, paraffin-embedded through the University of Michigan Orthopedic Research Laboratory Histology Core facility, and sectioned at 5 μm thickness. Sections were stained with hematoxylin and eosin as previously described (65) or with Picrosirius red according to manufacturer protocol (24901, Polysciences). Sections were imaged using an inverted ZEISS microscope with a 20× objective.

### Picrosirius red quantification.

Picrosirius red–stained liver sections were analyzed using the Aivia AI-based image analysis software (Leica Microsystems, Inc.). With the guidance of the Michigan Diabetes Research Center Microscopy and Image Analysis Core, Pixel Classifier profiles in Aivia were trained to segment collagen fibers from normal liver tissue. Once the pixel training was optimal, areas of Picrosirius-positive staining were quantified from the images with Aivia’s Recipe Console tool. At least 3 sections per mouse were counted and averaged.

### Quantitative PCR for AAV transduction.

DNA was isolated from psWAT, BAT, and liver using the QIAGEN Puregene Kit. DNA concentrations were normalized to 30 ng/μL, and primers targeting the woodchuck hepatitis virus posttranscriptional regulatory element (FW: 5′-CTTCCCGTATGGCTTTCATTTT-3′, RV: 5′-GCCGTGGCAAGAACTAACCA-3′) were used to amplify DNA with qPCRBIO SyGreen Mix (Innovative Solutions), then detected with a StepOnePlus System (Applied Biosystems). All primers were validated with DNA titration curves prior to use; quantitative PCR product specificities were confirmed by melting curve analysis. Gene expression was calculated using a DNA titration curve within each plate, normalized to reference gene *Hprt* (FW: 5′-TCATTATGCCGAGGATTTGGA-3′, RV: 5′-GCACACAGAGGGCCACAAT-3′), and calculated as change relative to control samples.

### Liver RNA-Seq.

Mouse livers were powdered at –80°C on dry ice, and 50 mg was homogenized in 1 mL RNA STAT-60 (CS110, AMSBIO) on ice. Samples were spun at 12,000*g* for 10 minutes at 4°C to pellet tissue debris. The clear supernatant was pipetted into a new tube, and 200 μL of chloroform was added to the tube and inverted. The samples were spun at 12,000*g* for 10 minutes at 4°C, and the aqueous supernatant was moved to a new tube. Isopropanol was added in equal volume to precipitate RNA overnight at –20°C. Tubes were spun at 12,000*g* for 10 minutes at 4°C to isolate precipitated RNA, and supernatant was removed. RNA pellet was washed with 75% ethanol 3 times. After a final spin at 12,000*g* for 10 minutes at 4°C, the ethanol wash was removed, and the RNA pellet was air-dried for 3 minutes. RNA was resuspended in diethylpyrocarbonate (DEPC)-treated water and quantified via NanoDrop spectrophotometer (Thermo Fisher Scientific).

Samples were sequenced by the University of Michigan Advanced Genomics Core, with libraries constructed and subsequently subjected to 150 paired-end cycles on the NovaSeq 6000 platform (Illumina). Data were prefiltered to remove genes with 0 counts in all samples. Differential gene expression analysis was performed using DESeq2 (66), using a negative binomial generalized linear model (thresholds: linear fold change >1.5 or <–1.5, Benjamini-Hochberg FDR [Padj] < 0.05). Annotation data from ENSEMBL 102 were used, and genes were additionally annotated with Entrez Gene IDs and text descriptions. Functional analysis, including candidate pathways activated or inhibited in comparison(s) and Gene Ontology term enrichments (67), was performed using GSEA (68).

### Bone static histomorphometry.

Tibias of 7-month-old female and male mice were dissected and fixed in 10% formalin (Thermo Fisher Scientific) for 24 hours at 4°C, dehydrated with graded concentrations of ethanol, and processed for nanoCT scanning. For tibial trabecular parameters, a region of interest (ROI) was defined starting at 0.75 mm proximal to the growth plate of femurs and extending 2 mm in the direction of the diaphysis where trabeculae were no longer visible. For tibial cortical parameters, ROI was defined starting at 0.25 mm above the tibia/fibula junction landmark. A region of 0.5 mm in height at midshaft was analyzed. The data analysis and images were generated using Dragonfly software (3D), version 2024 (Comet Technologies Canada Inc.).

### BMAT quantification by osmium staining.

Tibias of 7-month-old female mice were stained with osmium tetroxide for marrow fat analysis as previously described (69). Intact tibias were fixed in 10% formalin for 24 hours at 4°C (Thermo Fisher Scientific) and processed for decalcification in 14% EDTA, pH 7.4, for 14 days. Samples were then transferred to Sorensen’s phosphate buffer pH 7.2 in 1.5 mL microtubes. All subsequent steps were performed in the fume hood. Tibias were stained in 1% osmium tetroxide solution (Electron Microscopy Services) and incubated for 48 hours at room temperature on a rotating wheel. Stained tibias were then washed with Sorensen’s buffer twice for 3 hours and then overnight. Stained tibias were embedded in 1% agarose and processed for scanning. Quantification of marrow adipose tissue volume included the ROIs starting at the growth plate to the tibia/fibula junction (proximal) and tibia/fibula junction to the distal end of the bone (distal). The data analysis and images were generated using Dragonfly software (3D), version 2024.

### NanoCT analysis.

For nanoCT, tibias were immobilized inside a plastic fixture and scanned using a Phoenix Nanotom M nanoCT (Waygate Technologies, Baker Hughes). The x-ray tube was powered to 80 kV and 400 μA, utilized a diamond-coated tungsten target and a 0.381 mm aluminum filter, and was set to a spot size of 0. Imaging was done at either 10 μm or 8 μm voxel size using an exposure time of 500 ms, with 3 frames averaged and 1 skipped for each rotation. The sample stage rotated through 360 degrees and collected 1,000 images per scan. Image acquisition and reconstruction of raw data were performed using Datos x 2 version 2.6.1 (Waygate Technologies, Baker Hughes).

### Indirect calorimetry.

Mice were housed individually and continuously fed a 60% HFD for 3 days, and then the mice were placed into an indirect calorimetry system (Promethion Core, Sable Systems International), where their O_2_ consumption and CO_2_ production, in-cage activities, and food and water intake were measured at room temperature (22°C, 41% humidity) for 3 days (12–12 hours 5:00pm~5:00am dark/light cycles). All animals were continuously maintained on the 60% HFD throughout the experiment and had free access to food and water. Oxygen consumption (VO_2_), carbon dioxide production (VCO_2_), and spontaneous motor activity were measured using the Promethion system, an integrated open-circuit calorimeter equipped with an optical beam activity-monitoring device. The Promethion system features high-precision sensors measuring food and water intake and body weight in real time. The system was routinely calibrated each time before the experiment using a standard gas (20.5% O_2_ and 0.5% CO_2_ in N_2_). VO_2_ and VCO_2_ in each cage were sampled sequentially for 30 seconds in 5-minute intervals, and the motor activity was recorded every second in *X*, *Y*, and *Z* dimensions. The air flow rate through the chambers was adjusted at the level to keep the oxygen differential around 0.3% at resting conditions. Respiratory exchange ratio was calculated as VCO_2_/VO_2_. Total energy expenditure, carbohydrate oxidation, and fatty acid oxidation were calculated respectively based on the values of VO_2_ and VCO_2_, without the estimation of protein breakdown (which is usually estimated from urinary nitrogen excretion) (70–76).

### Statistics.

All data were presented as mean ± SD unless noted in the figure legend. Two-way ANOVA with Bonferroni’s post hoc test was performed for all data presented in the study. All analyses were conducted using GraphPad Prism version 10. For statistical comparisons, *P* < 0.05 was considered significant.

### Study approval.

All animal studies were performed in compliance with policies and with the approval of the University of Michigan Institutional Animal Care and Use Committee (Protocol PRO00011544).

### Data availability.

A Supporting Data Values file is contained in the supplement. Liver RNA-Seq data are available through NCBI GEO (accession no. GSE294358).

## Author contributions

JNM, YC, CDS, ND, and OAM conceived the studies and planned the experimental design. JNM, YC, KSH, REA, HH, MJD, TAH, GAF, RLS, HM, RMU, DG, KTL, IDKH, CDS, and AT performed the experiments. JNM, YC, KSH, HH, CDS, EAO, ND, and OAM analyzed the data. JNM, YC, and OAM wrote the manuscript, while all other authors edited and approved the final manuscript. JNM and YC are co–first authors, and authorship order was assigned based on project leadership.

## Supplementary Material

Supplemental data

Unedited blot and gel images

Supporting data values

## Version 1 07/15/2025

In-Press Preview

## Version 2 08/22/2025

Electronic publication
