# Supplementary material for: Effects of FGF21, soluble TGFBR2, and environmental temperature on metabolic dysfunction in lipodystrophic mice
Source: JCI Insight. 2025 Jul 15;10(16):e194882. doi: 10.1172/jci.insight.194882 (PMC12406727; doi:10.1172/jci.insight.194882)
Supplement: Supplemental data [file jciinsight-10-194882-s232.pdf]

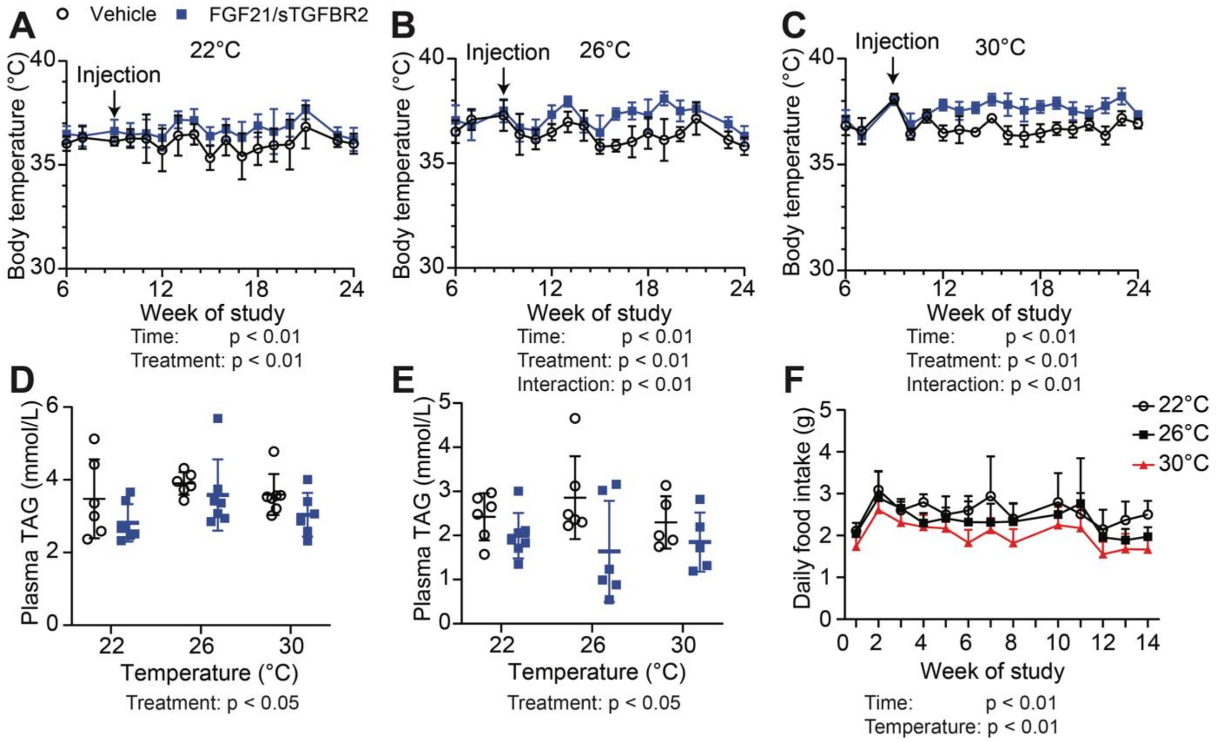

Figure S1. FGF21/sTGFBR2 reduces plasma TAG concentrations and increases body temperature in high fat diet-fed mice. Core body temperature over time in HFD-fed female mice housed at (A) 22°C, (B) 26°C and (C) 30°C (n = 6-7). Plasma TAG concentrations at (D) four weeks and (E) six weeks post-treatment (n = 6-7). (F) Daily food intake in mice at different housing temperatures. Statistical analyses were performed using two-way ANOVA, followed by Bonferroni's post-hoc test.

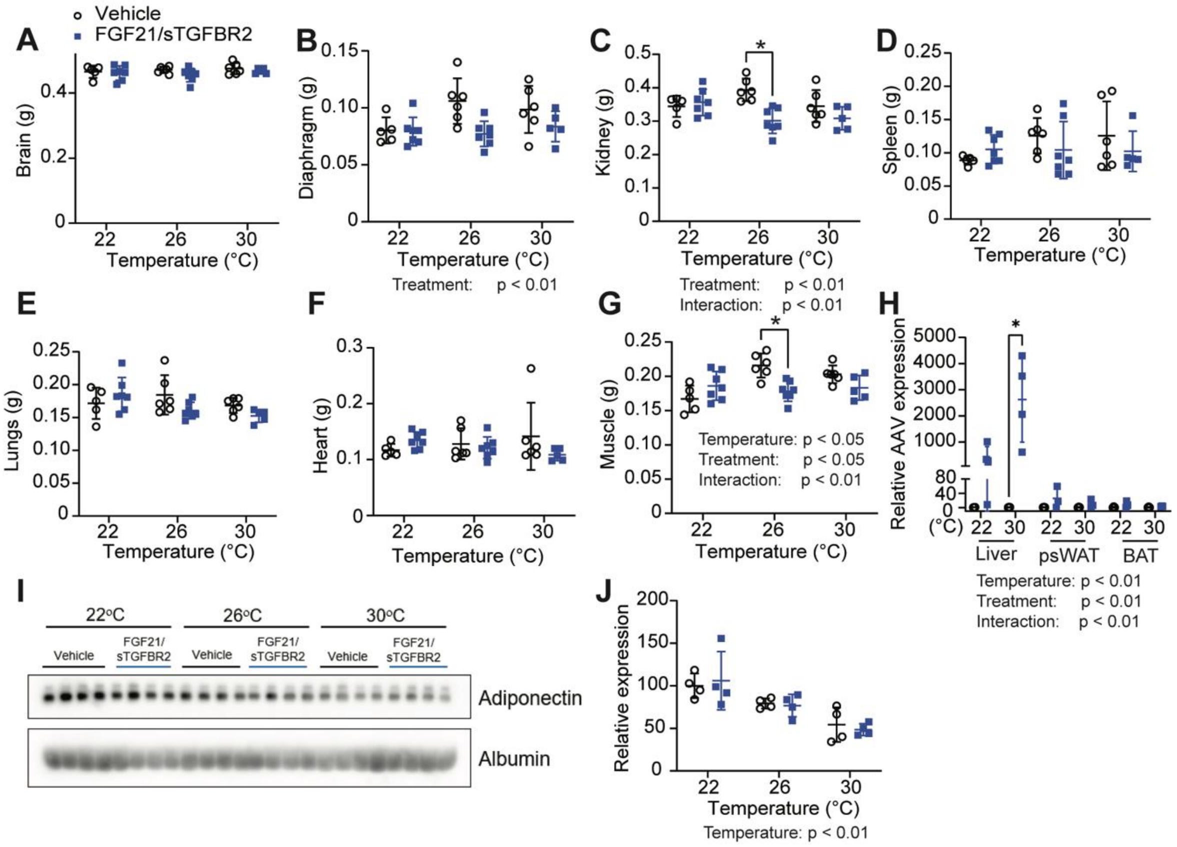

Figure S2. FGF21/sTGFBR2 decreases diaphragm weights and kidney weights in HFD-induced obese mice. Female mice were euthanized ten weeks after FGF21/sTGFBR2 treatment (n = 5-7). Weights of (A) brain, (B) diaphragm, (C) kidneys, (D) spleen, (E) lungs, (F) heart, and (G) gastrocnemius muscle. (H) Quantification of AAV transduction in liver, psWAT, and BAT. (I) Plasma adiponectin immunoblot and (J) quantification relative to controls housed at 22°C. Statistical analyses were performed using two-way ANOVA, followed by Bonferroni's post-hoc test.

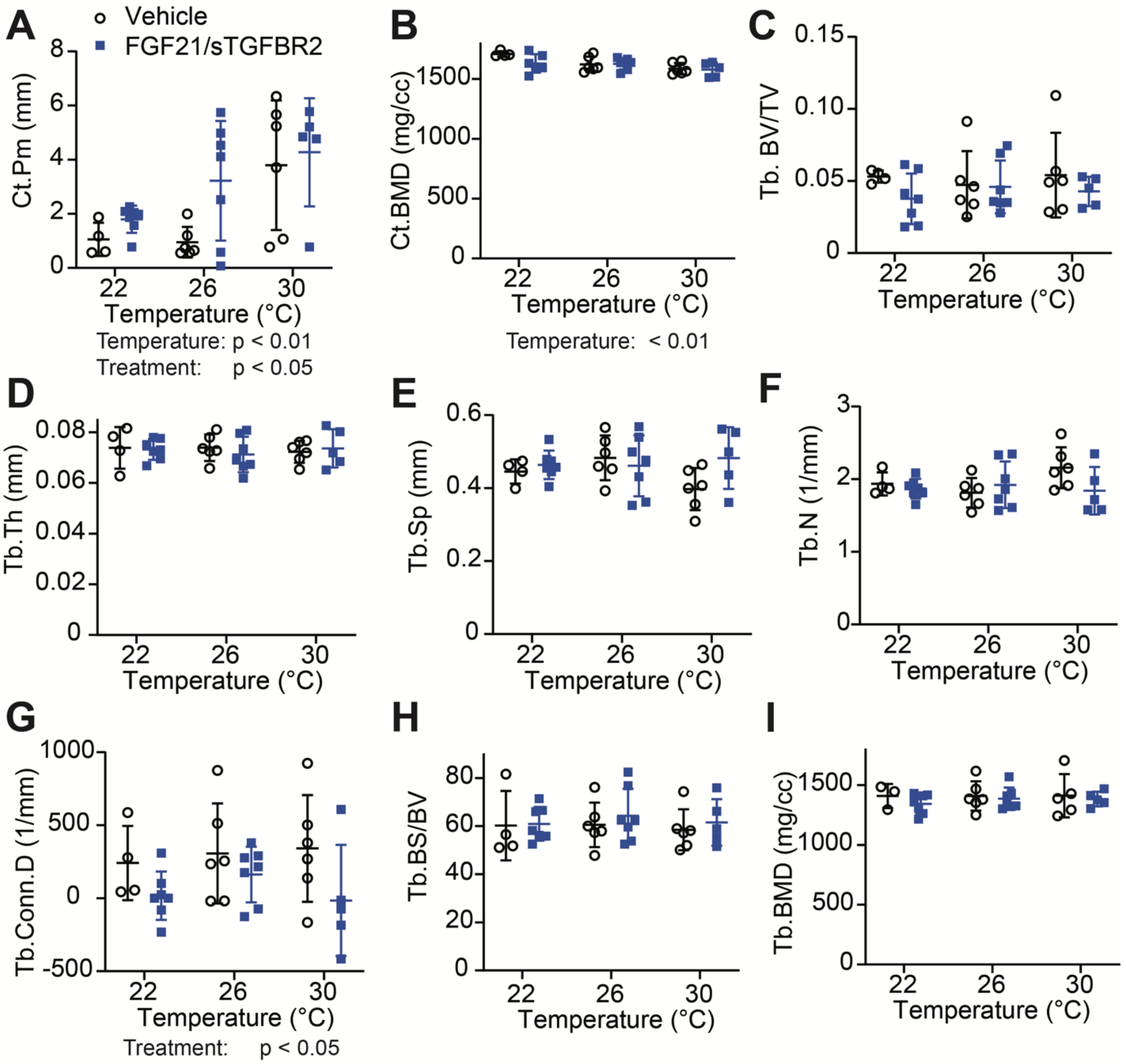

Figure S3. FGF21/sTGFBR2 decreases tibial trabecular connectivity density and increases cortical perimeter. Female mice were euthanized ten weeks after FGF21/sTGFBR2 transduction (n = 4-7). Tibias were analyzed by nanoCT. (A) Cortical perimeter (Ct.Pm). (B) Cortical bone mineral density (Bt.BMD). (C) Trabecular bone volume fraction (Tb. BV/TV). (D) Trabecular thickness (Tb.Th). (E) Trabecular separation (Tb.Sp). (F) Trabecular number (Tb.N). (G) Trabecular connectivity density (Tb.Conn.D). (H) Trabecular specific bone surface

(Tb.BS/BV). (I) Trabecular bone mineral density (Tb.BMD). *P < 0.05. Statistical analyses were performed using two-way ANOVA, followed by Bonferroni's post-hoc test.

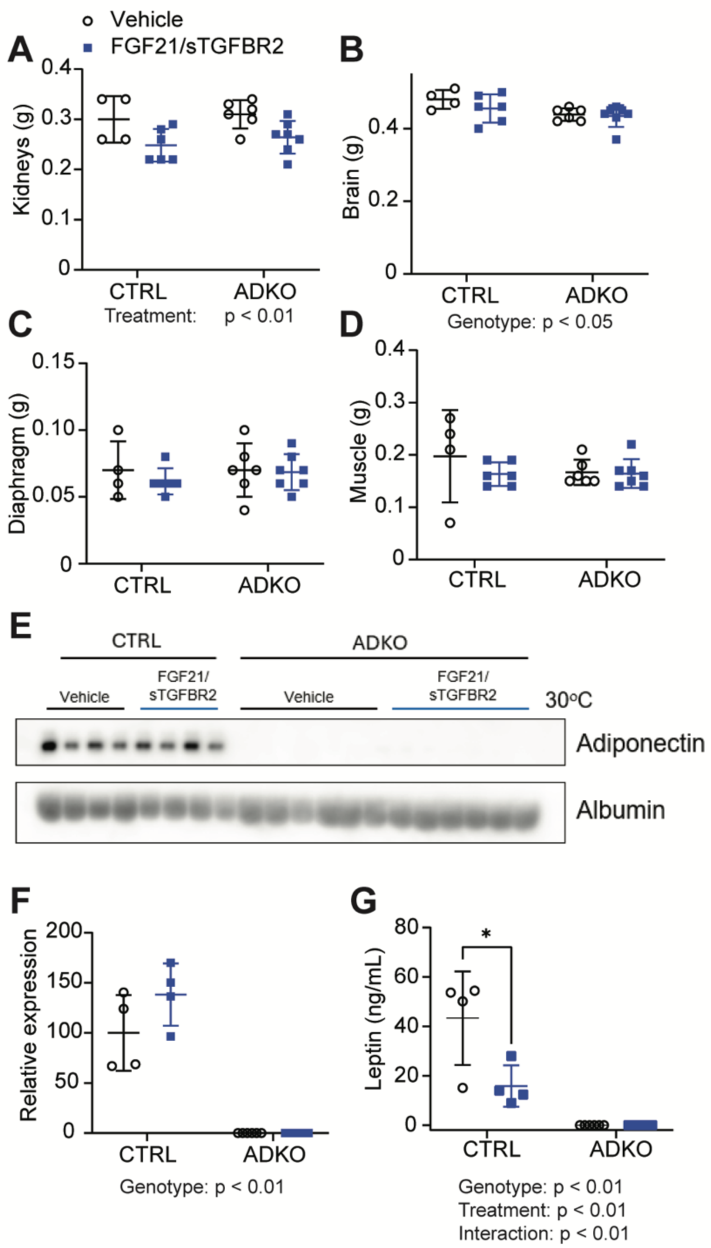

Figure S4. FGF21/sTGFBR2 decreases kidney weights in lipodystrophic mice housed at 30°C. Female mice were euthanized 12 weeks after transduction with FGF21/sTGFBR2 (n = 4-7). Weights of (A) kidneys, (B) brain, (C) diaphragm, and (D) gastrocnemius muscle at euthanasia. (E) Immunoblot of plasma adiponectin with (F) quantification. (G) Fed leptin concentrations at euthanasia. *$P < 0.05$. Statistical analyses were performed using two-way ANOVA, followed by Bonferroni's post-hoc test.

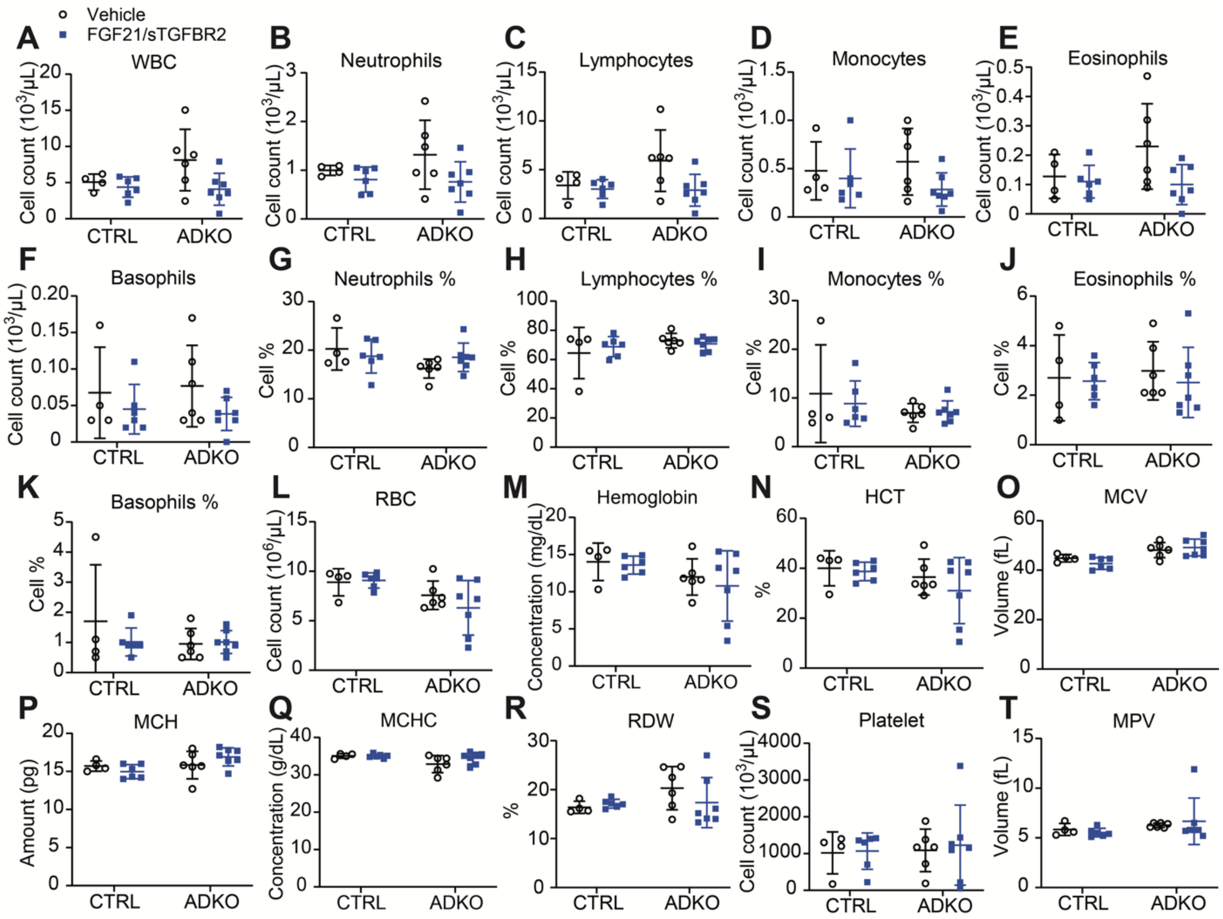

Figure S5. FGF21/sTGFBR2 does not change complete blood counts in lipodystrophic mice. Blood was taken at euthanasia 12 weeks after transduction with FGF21/sTGFBR2 (n = 4-7). Total cell counts of (A) white blood cells, (B) neutrophils, (C) lymphocytes, (D) monocytes, (E) eosinophils, and (F) basophils. Cellular percentage of (G) neutrophils, (H) lymphocytes, (I) monocytes, (J) eosinophils, and (K) basophils. (L) Red blood cell count. (M) Hemoglobin concentrations. (N) Hematocrit (HCT). (O) Mean corpuscular volume (MCV). (P) Mean corpuscular hemoglobin (MCH) amount. (Q) Mean corpuscular hemoglobin concentrations. (R) Red blood cell distribution width. (S) Platelet count. (T) Mean platelet volume. Statistical analyses were performed using two-way ANOVA, followed by Bonferroni's post-hoc test.

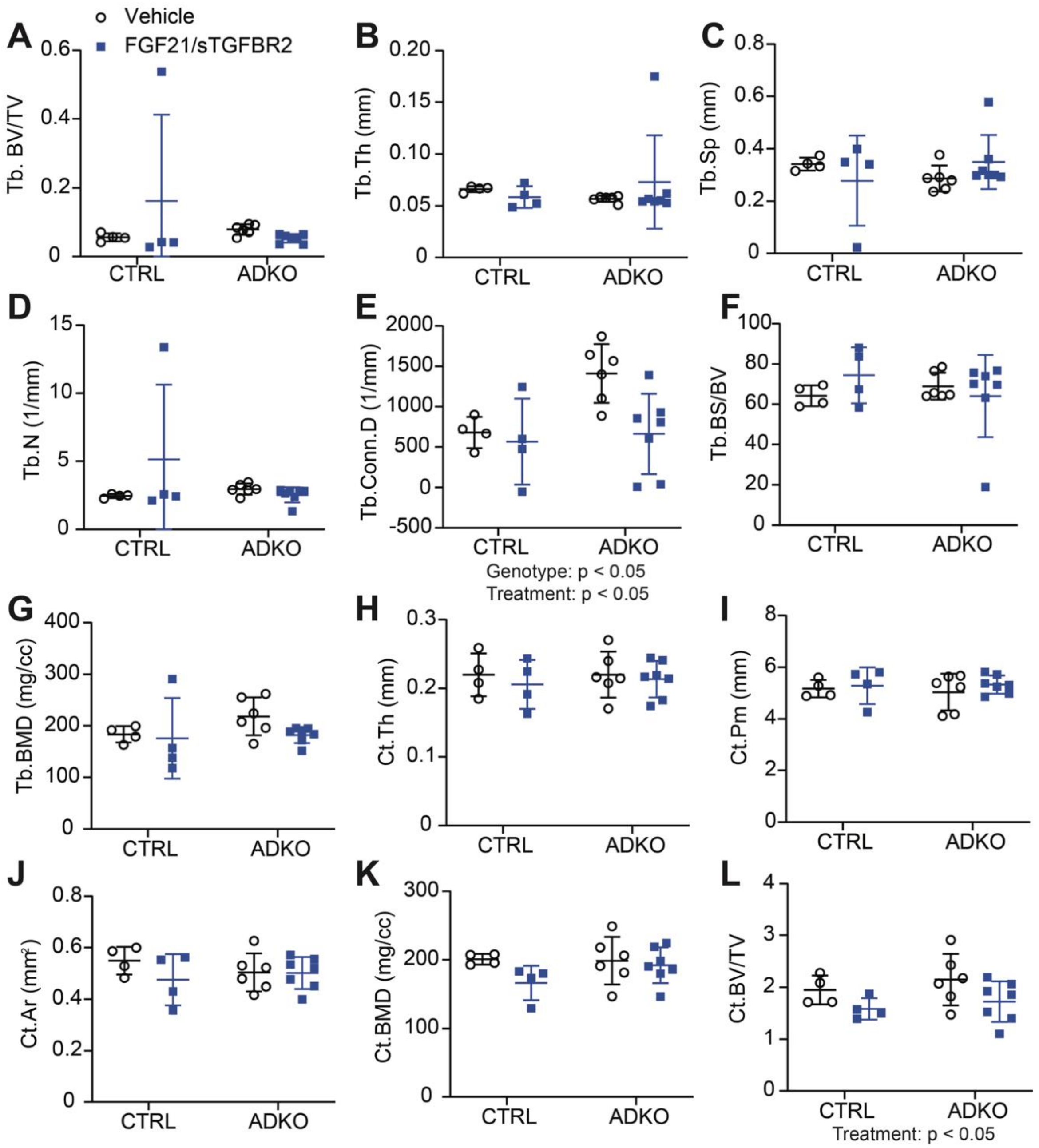

Fig S6. FGF21/sTGFBR2 decreases tibial trabecular connectivity density and cortical bone volume fraction in lipodystrophic mice housed at thermoneutrality. Female mice were euthanized ten weeks after FGF21/sTGFBR2 transduction (n = 4-7). Tibias were analyzed by nanoCT. (A) Trabecular bone volume fraction (Tb. BV/TV). (B) Trabecular thickness (Tb.Th). (C) Trabecular separation (Tb.Sp). (D) Trabecular number (Tb.N). (E) Trabecular connectivity density (Tb.Conn.D). (F) Trabecular specific bone surface (Tb.BS/BV). (G) Trabecular bone

mineral density (Tb.BMD). (H) Cortical thickness (Ct.Th). (I) Cortical perimeter (Ct.Pm). (J) Cortical area (Ct.Ar). (K) Cortical bone mineral density (Ct.BMD). (L) Cortical specific bone surface (Ct.BS/BV). Statistical analyses were performed using two-way ANOVA, followed by Bonferroni's post-hoc test.

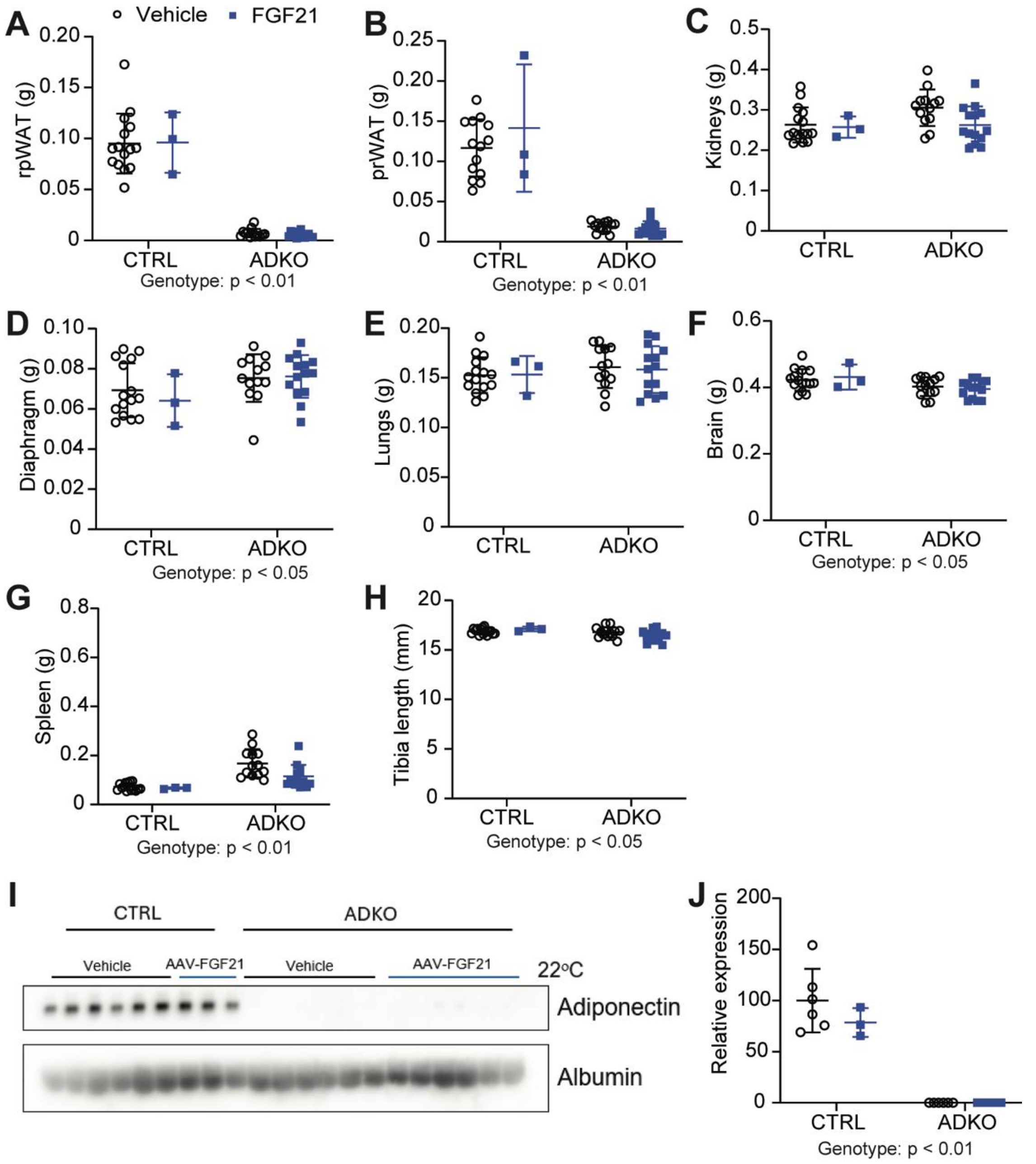

Figure S7. FGF21 has little effect on organ weights in lipodystrophic mice housed at 22°C. Female mice were euthanized eight weeks after FGF21 treatment (n = 3-15). Weights of (A) rpWAT, (B) prWAT, (C) kidneys, (D) diaphragm, (E) lungs, (F) brain, (G) spleen. (H) Tibia length. (I) Plasma adiponectin immunoblot with (J) quantification. Statistical analyses were performed using two-way ANOVA, followed by Bonferroni's post-hoc test.
