# Supplementary material for: Effects of FGF21, soluble TGFBR2, and environmental temperature on metabolic dysfunction in lipodystrophic mice
Source: JCI Insight. 2025 Jul 15;10(16):e194882. doi: 10.1172/jci.insight.194882 (PMC12406727; doi:10.1172/jci.insight.194882)

Unedited gel image for Figure S2I for adiponectin

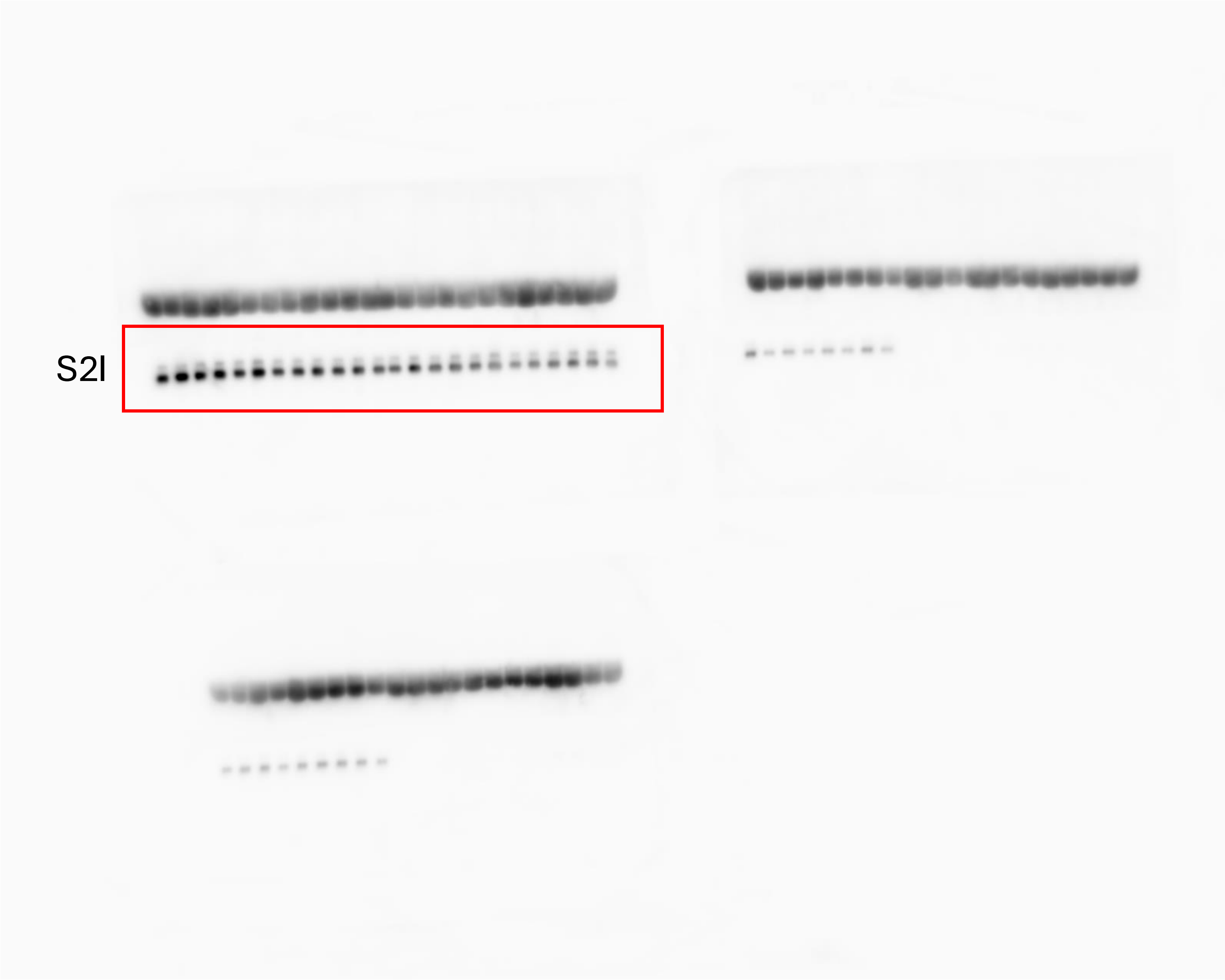

Unedited gel image for Figure S4E and S7I for adiponectin

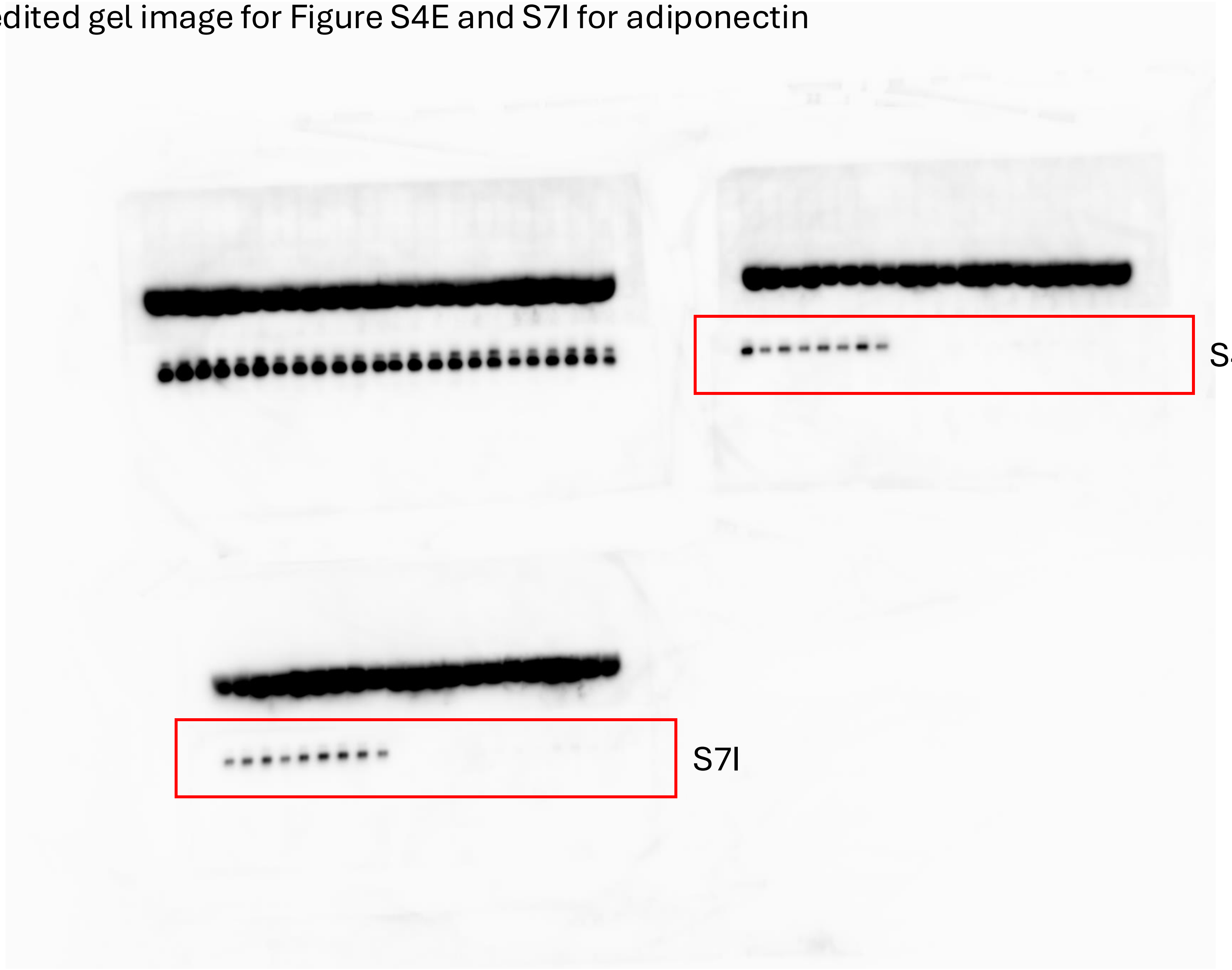

S4E

S7I

Unedited gel image for Figure S2I, S4E and S7I for albumin

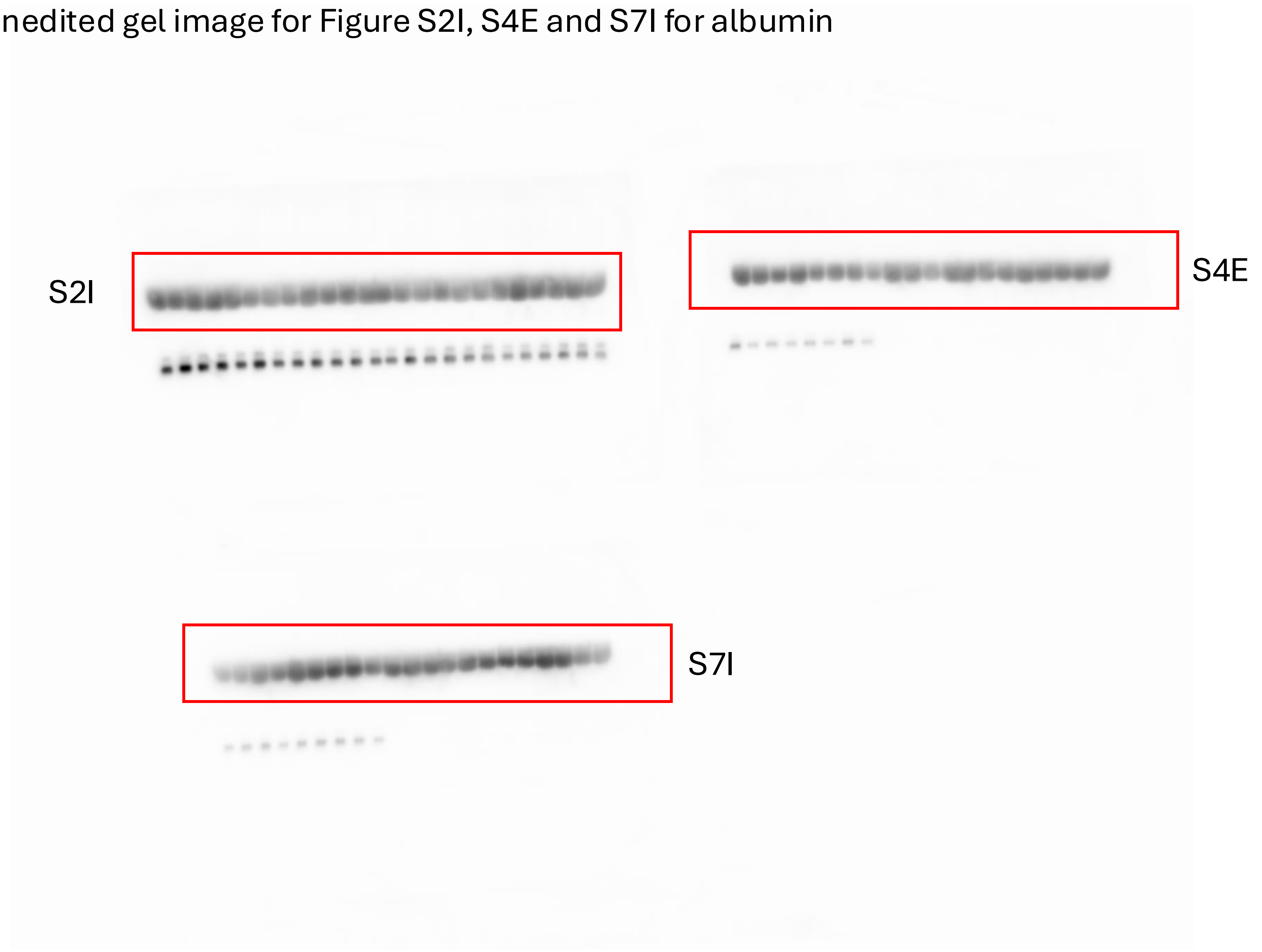

Unedited gel image for Figure S2I, S4E and S7I for albumin and adiponectin – showing gel markers

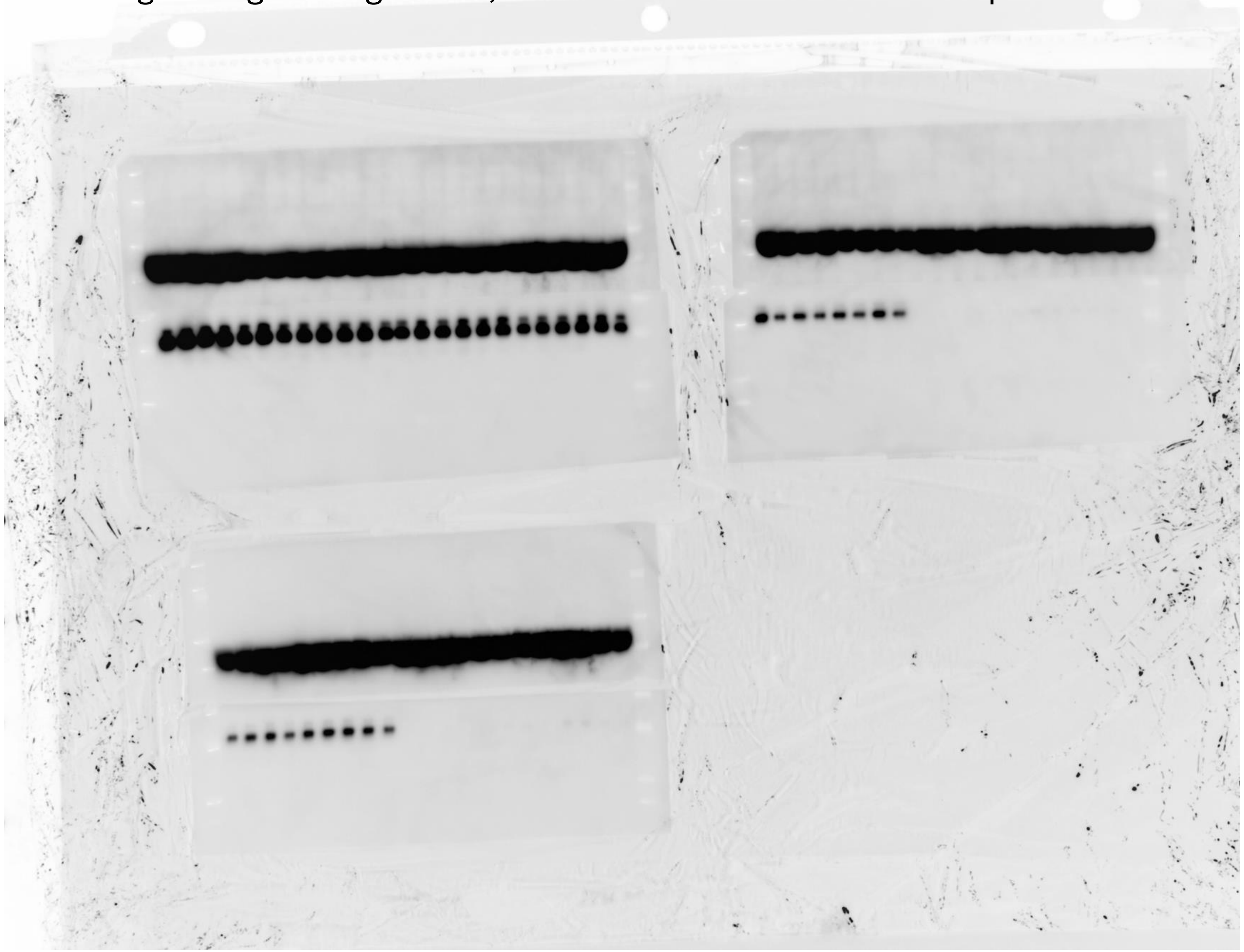

Supplement: Unedited blot and gel images [file jciinsight-10-194882-s233.pdf]
